# Association between post-COVID-19 neuropsychiatric symptoms and persistent glial activation in the limbic system: a TSPO PET study

**DOI:** 10.1007/s00415-026-13842-w

**Published:** 2026-04-30

**Authors:** Joel Tuomaala, Maija Saraste, Emma Smith, Matilda Kuusi, Elisabet Westerberg, Eveliina Honkonen, Rahim Kargar, Sini Laaksonen, Jussi Lehto, Amelie Luoma, Markus Matilainen, Olavi Misin, Janne Atosuo, Mari Kanerva, Helena Liira, Sini Laakso, Tatiana Posharina, Virva Saunavaara, Saara Wahlroos, Johan Rajander, Laura Airas

**Affiliations:** 1 Turku PET Centre, Turku University Hospital, University of Turku, and Åbo Akademi University, Turku, Finland; 2 Turku University Hospital, Neurocenter, Turku, Finland; 3 Clinical Neurosciences, University of Turku, Turku, Finland; 4 InFLAMES Research Flagship, University of Turku, Turku, Finland; 5 Åland Central Hospital, Mariehamn, Åland Finland; 6 Department of Biotechnology, University of Turku, Turku, Finland; 7 Department of Hospital Hygiene and Infection Control, TYKS Turku University Hospital, Turku, Finland; 8 Outpatient Clinic for Long-Term Effects of COVID-19, Helsinki University Central Hospital, and University of Helsinki, Helsinki, Finland; 9 Radiopharmaceutical Chemistry Laboratory, Turku PET Centre, University of Turku, Turku, Finland; 10 Accelerator Laboratory, Åbo Akademi University, Turku PET Centre, Turku, Finland

**Keywords:** [^11^C]PK11195, Glial activation, Limbic system, Long COVID, Neuropsychiatric symptoms, TSPO PET

## Abstract

**Background:**

A subset of individuals experience prolonged neurological and psychiatric symptoms following SARS-CoV-2 infection, a condition referred to as long COVID (LC). Limited evidence implicates ongoing neuroinflammatory processes as a driver of LC. This study investigates neuroinflammation in LC using translocator protein positron emission tomography (TSPO PET).

**Methods:**

14 LC, 11 healthy control (HC) and 13 multiple sclerosis (MS) participants were included in the study. They underwent [^11^C]PK11195 TSPO PET and 3T magnetic resonance imaging (MRI) to evaluate glial activation, white matter (WM) pathology and brain volumetrics. Serum neurofilament light chain (NfL) and glial fibrillary acidic protein (GFAP) were measured as markers of neuronal and glial damage. LC participants completed neurological examinations and mental health assessments.

**Results:**

TSPO availability, measured as distribution volume ratio (DVR), was not elevated in LC compared to HCs but was significantly lower in LC compared to MS (WM DVR 1.03 vs. 1.06; *p* = 0.007). Individuals imaged within 16 months of SARS-CoV-2 infection showed higher WM DVR compared to those with a longer disease duration (1.05 vs. 1.02; *p* = 0.04). Moreover, lower quality of life was associated with higher DVRs in the hippocampus, amygdala and thalamus (ρ = − 0.83- − 0.70), and depression and anxiety correlated positively with DVRs in the hippocampus and amygdala (ρ = 0.75–0.97).

**Conclusions:**

LC TSPO availability did not differ from HCs in any studied brain area. However, lower WM TSPO availability in individuals with longer LC duration suggests COVID-19-associated neuroinflammation may subside with time, while the association between limbic TSPO availability and LC severity may imply a role for limbic activity in LC symptomology.

**Supplementary Information:**

The online version contains supplementary material available at 10.1007/s00415-026-13842-w.

## Introduction

In the aftermath of the COVID-19 pandemic, a substantial proportion of individuals report a variety of persistent or new-onset symptoms, collectively referred to as long COVID (LC). The World Health Organisation (WHO) defines LC as the continuation or development of new symptoms 3 months after the initial SARS-CoV-2 infection, with these symptoms lasting for at least 2 months with no other explanation [1]. The commonly reported symptoms, such as fatigue, post-exertional malaise, difficulties in concentrating, headaches, depression and anxiety [2, 3], suggest possible post-infectious cerebral involvement. Emerging evidence implicates neuroinflammation as a key contributor [4, 5], but also other mechanisms such as damage to blood vessels, neuronal injury and autoimmune response against the nervous system have been suggested [6, 7].

Positron emission tomography (PET) imaging using 18-kDa translocator protein (TSPO)-specific radioligands can detect glial activation, a hallmark of neuroinflammation [8]. This technique is widely used in the study of neuroinflammatory conditions such as multiple sclerosis (MS). In the context of LC, increased TSPO availability has been found in multiple brain regions along with diverse symptom profiles [9]. Further, elevated TSPO total distribution volume (Vt) was also reported in people who had developed depression following a COVID-19 infection [10].

Serum glial fibrillary acidic protein (GFAP) and neurofilament light chain (NfL) are biomarkers which reflect astrocytic and axonal damage, respectively [11]. During acute COVID-19, both GFAP and NfL can be elevated [12], particularly in patients exhibiting neurological symptoms [13]. At approximately 3 months post-infection, increased GFAP (but not NfL) levels have been reported in those with neurological LC symptoms; however, by 6 months post-infection, levels of both markers typically return to baseline, irrespective of symptom persistence [14–17].

This study investigates whether neuroinflammation is elevated in the context of prolonged neurological and psychiatric LC symptoms by quantifying glial activation via TSPO PET imaging using the [^11^C]PK11195 radioligand. Additionally, we assess serum levels of GFAP and NfL and examine their correlation with PET-derived measures of neuroinflammation. Finally, as an exploratory analysis, we evaluate associations between TSPO binding and participant-reported disability, wellbeing, and neuropsychiatric and cognitive symptoms.

## Materials and methods

### Participants and study design

People with LC symptoms were prospectively recruited from the Neurocenter Outpatient Clinic of Turku University Hospital, Turku, Finland and from the Long COVID Outpatient Clinic at Helsinki University Hospital, Helsinki, Finland in 2021–2023. Inclusion criteria were age ≥ 18 years, neurological symptoms like fatigue or cognitive symptoms that interfered with work or study capacity, symptoms that had lasted for at least 2 months and were present at 3 months after the SARS-CoV-2 infection, and a confirmation of COVID-19 condition by a positive polymerase chain reaction (PCR) or antigen test during the acute infection phase. The exclusion criteria for LC participants were another condition causing symptoms associated with LC, pregnancy or breastfeeding, corticosteroid treatment within the past 4 weeks before PET/MRI, claustrophobia or history of severe anxiety or panic attacks, exposure to experimental radiation within the past 12 months, and intolerance to PET or MRI scans.

Age- and sex-matched HCs with no known neurological symptoms or diseases and patients with MS were used as control groups for imaging parameters and biomarker assays. HCs were prospectively recruited for this study from Turku University Hospital staff and members of the local community through staff networks (2021–2022). MS participants were originally recruited for other studies at the MS Neurocenter Outpatient Clinic of Turku University Hospital based on a confirmed MS diagnosis using current diagnostic criteria and willingness to participate in PET studies (2019–2022) and were retrospectively included in this study. The MS PET data presented in this study have not been used in other publications.

All study participants underwent PET and MRI scans and blood sampling. Clinical neurological examination was performed for individuals with LC for assessment of neurological symptoms and for patients with MS to assess the Expanded Disability Status Scale (EDSS) scores. The LC participants freely self-reported unstructured descriptions of their medical history, LC symptoms and hospitalisation details related to COVID-19 during a medical consultation and answered several mental health questionnaires.

Imaging parameters and biomarker assays were compared between the LC, MS, and HC groups to determine whether LC is associated with overall elevated neuroinflammation relative to a healthy reference group and a neuroinflammatory disease control. Mental health questionnaires were completed by LC participants only, while the HC and MS groups served exclusively as imaging and biomarker reference groups. Within the LC group, associations between questionnaire scores and imaging parameters were examined in an exploratory manner to evaluate the relationship between subjective symptom severity and neuroinflammation.

### Questionnaires

LC participants filled in Finnish versions of two quality of life questionnaires (European Health Interview Survey—Quality of Life [EuroHIS-8], RAND Corporation 36-item Short Form Health Survey [RAND SF-36]) [18, 19], two fatigue questionnaires (Fatigue Severity Scale [FSS], Modified Fatigue Impact Scale [MFIS]) [20, 21], anxiety (General Anxiety Disorder-7 [GAD-7]) [22] and depression questionnaires (Patient Health Questionnaire 9 [PHQ-9]) [23], insomnia (Insomnia Severity Index [ISI]) [24] and finally, health and disability questionnaires (WHO Disability Assessment Schedule 2.0 [WHODAS 2.0]) [25]. Questionnaire scores were calculated according to instructions as either the average of the total scores (EuroHIS-8, FSS), the sum of the total scores (MFIS, GAD-7, PHQ-9, ISI, and WHODAS 2.0) or the average of subsegments (RAND SF-36). For HC and MS participants, questionnaires were not obtained.

### Biomarker analysis

Serum NfL and GFAP concentrations were measured with a single molecule array assay (Neurology 2 plex B, Simoa® Technology, Quanterix, Billerica, MA, U.S.) from serum samples collected as previously described [26].

### PET/MR image acquisition and processing

The PET/MR imaging was performed at the Turku PET Centre (Turku, Finland) with GE SIGNA™ PET/MR scanner (GE HealthCare, Chicago, IL, U.S.). Beginning at radioligand injection, a 30-min 3.0 T brain MRI and a dynamic 60-min whole brain [^11^C]-PK11195 PET were performed simultaneously. MRI included axial T2, 3D fluid-attenuated inversion recovery (FLAIR) and 3D T1 sequences. The [^11^C]PK11195 radioligand radiochemical synthesis was performed as previously described [27]. The mean (SD) injected radioligand was 443.5 (63.4) for HC, 445.4 (47.15) for MS, and 429.6 (56.3) for LC participants, with no significant differences between groups.

T1 MR images were used for auto segmentation of regions of interest (ROIs) with the FreeSurfer image analysis suite v7.2.0. (http://surfer.nmr.mgh.harvard.edu/). ROIs used in this study were the whole brain, white matter (WM), cortical gray matter (GM), amygdala, hippocampus, thalamus, putamen, pallidum, cingulate cortex, corpus callosum and brain stem. In the MS participants, the WM ROI was converted to a normal-appearing white matter (NAWM) ROI by subtracting manually curated MS lesion masks from it. For the LC and HC participants, the WM and NAWM ROIs were identical. ROI volumes were calculated from MR images based on segments created in FreeSurfer as described previously [27]. Intracranial volumes (ICVs) were calculated using SPM12. ROI volumes are reported as parenchymal fractions (PF), defined as the ratio of brain region parenchymal volume to total intracranial volume.

MR and PET image co-registration was performed using SPM12 (Wellcome Trust Center for Neuroimaging) running on Matlab 2017a (The MathWorks, Natick, MA, U.S.). PET images were reconstructed using Bayesian penalised likelihood algorithm Q.Clear that incorporates a time-of-flight model and point spread function correction. A penalization factor β = 350, 17 time frames, and voxel dimensions 1.172 × 1.172 × 2.78 mm (radial, tangential, axial) were used for reconstruction. Mutual information realignment in SPM12 was used to correct possible displacements between frames. PET had an approximate spatial resolution of 4.4 × 4.1 × 5.5 mm as determined by NEMA 2012 1 cm sphere standard test with ^18^F isotope. All images were resliced to the MRI voxel size of 1 × 1 × 1 mm. Specific binding of [^11^C]PK11195 radioligand was quantified as distribution volume ratio (DVR) in different ROIs using the Logan method within a time interval of 20–60 min. A supervised clustering algorithm with four predefined kinetic tissue classes was used with the Matlab SuperPK software package to acquire the time activity curve corresponding to a reference region devoid of specific radioligand binding [28, 29]. The kinetic classes describing binding in normal GM (GM cortex), normal WM (centrum semiovale), and the blood activity (95% threshold mask from the sum from the first five PET frames) were defined using data from the HCs. The kinetic class for the high-binding GM was extracted from the core of the thalamus of the participants with secondary progressive MS.

### Statistical analyses

Statistical analyses were conducted in R programming environment (R v4.3.2). Non-normality of data was determined using Shapiro–Wilk test (stats v4.3.2, shapiro.test), histograms and quantile–quantile plots (ggplot2 v3.5.1, geom_qq). For parameters non-normally distributed within any group, the median with first and third quantile limits (Q1–Q3) and Wilcoxon test (stats v4.3.2, wilcox.test) for group-wise comparisons is reported. Otherwise mean with standard deviation and two-sample Welch’s *t*-test (stats v4.3.2, *t*.test) is reported. In group-wise comparisons of sex proportions Fisher’s exact test (stats v4.3.2, fisher.test) is reported. Corrected *p*-values were adjusted for within-parameter multiple comparisons using Benjamini–Hochberg method (stats v4.3.2, p.adjust). For correlation analyses, Spearman correlation coefficient (ρ), 95% confidence interval and raw p-value are reported (psych v2.4.6.26, corr.test). Trendlines shown in correlation scatterplots are based on linear regression and are not derived from the associated Spearman correlation coefficients. Enrichment of significant correlations in limbic regions was assessed using an exact permutation test, enumerating all 165 possible combinations of 3 ROIs from the 11 examined. Analysis was restricted to non-dependent symptom scores WHODAS total, MFIS total, RAND-36 subscales, FSS total, GAD-7 total, PHQ-9 total, ISI total, and EUROHIS-QOL total to ensure independence of symptom measures. Fold enrichment was calculated relative to the null expectation of random proportional distribution across all 11 ROIs. Cronbach’s alpha test (psych v2.4.6.26, alpha) for covariance and Spearman–Brown corrected mean even–odd split-half reliability (psych v2.4.6.26, splitHalf) of all possible splits for internal reliability consistency among questionnaire replies were calculated for final and subsection scores across the corresponding individual questions. All tests were two-tailed and p-values under 0.05 were considered statistically significant for all analyses.

## Results

### Demographic data

14 participants with LC symptoms, 11 HC participants and 13 MS participants were included in the study. Six of the 14 LC participants had been hospitalised during the acute SARS-CoV-2 infection but did not require intensive care. In the HC group, five participants had been diagnosed with and had fully recovered from COVID-19 and were free of any LC-like symptoms prior to study participation. The recovered controls did not differ from other controls in demographic, serum biomarker or PET parameters (Table S1). The MS group contained six relapsing–remitting MS (RRMS) and seven secondary progressive MS (SPMS) patients with a median disease duration of 20 years and a median EDSS of 3.5. Age and weight did not differ significantly between the LC, MS and HC groups. The mean BMI of the LC participants was higher compared to HCs, but the difference was not significant after multiple comparison correction (Table 1).

**Table 1:** Demographic and soluble biomarker data of the study cohort

	MS	HC	LC	HC vs MS	LC vs MS	HC vs LC
*n*	13	11	14	–	–	–
*n* male/*n* female (% female)	3/10 (78)	7/4 (36)	8/6 (43)	0.095 (0.180)	0.120 (0.180)	1.000 (1.000)
Age (years)	46.79 (6.62)	45.55 (12.16)	45.00 (7.75)	0.766 (0.899)	0.524 (0.899)	0.899 (0.899)
Weight (kg)	85.00 (66.60–96.00)	78.90 (77.20–85.15)	102.10 (81.23–121.75)	0.728 (0.728)	0.152 (0.228)	0.095 (0.228)
BMI (kg/m^2^)	30.25 (8.37)	26.91 (3.89)	32.68 (7.23)	0.215 (0.322)	0.431 (0.431)	**0.019** (0.056)
NfL (pg/ml)	8.27 (5.28–14.12)	7.86 (4.31–9.59)	5.87 (4.97–10.29)	0.418 (0.704)	0.470 (0.704)	0.976 (0.976)
GFAP (pg/ml)	75.84 (64.49–124.65)	53.71 (44.41–63.17)	67.67 (55.07–89.61)	**0.043** (0.128)	0.347 (0.347)	0.343 (0.347)
Long COVID duration (months)	–	–	15.00 (10.00–23.50)	–	–	–
MS duration (years)	20.01 (18.30–24.44)	–	–	–	–	–
EDSS	3.50 (2.50–5.00)	–	–	–	–	–

Number of male and female participants, percentage of females, and Fisher’s exact test for group-wise comparisons are reported for sex. For other, normally distributed parameters, mean (SD) and t-test p-values are reported; for non-normally distributed parameters, median (Q1–Q3) and Wilcoxon test *p*-values are reported. *P*-values in brackets are corrected for within-parameter multiple comparisons using the Benjamini–Hochberg method. Significant *p*-values are bolded. *BMI* body mass index, *EDSS* Expanded disability status scale, *GFAP* glial fibrillary acidic protein, *HC* healthy control, *LC* long COVID, *MS* multiple sclerosis, *NfL* neurofilament light chain

### Self-reported neurological symptoms correspond poorly with clinical examination findings in long COVID participants

**Fig. 1:**
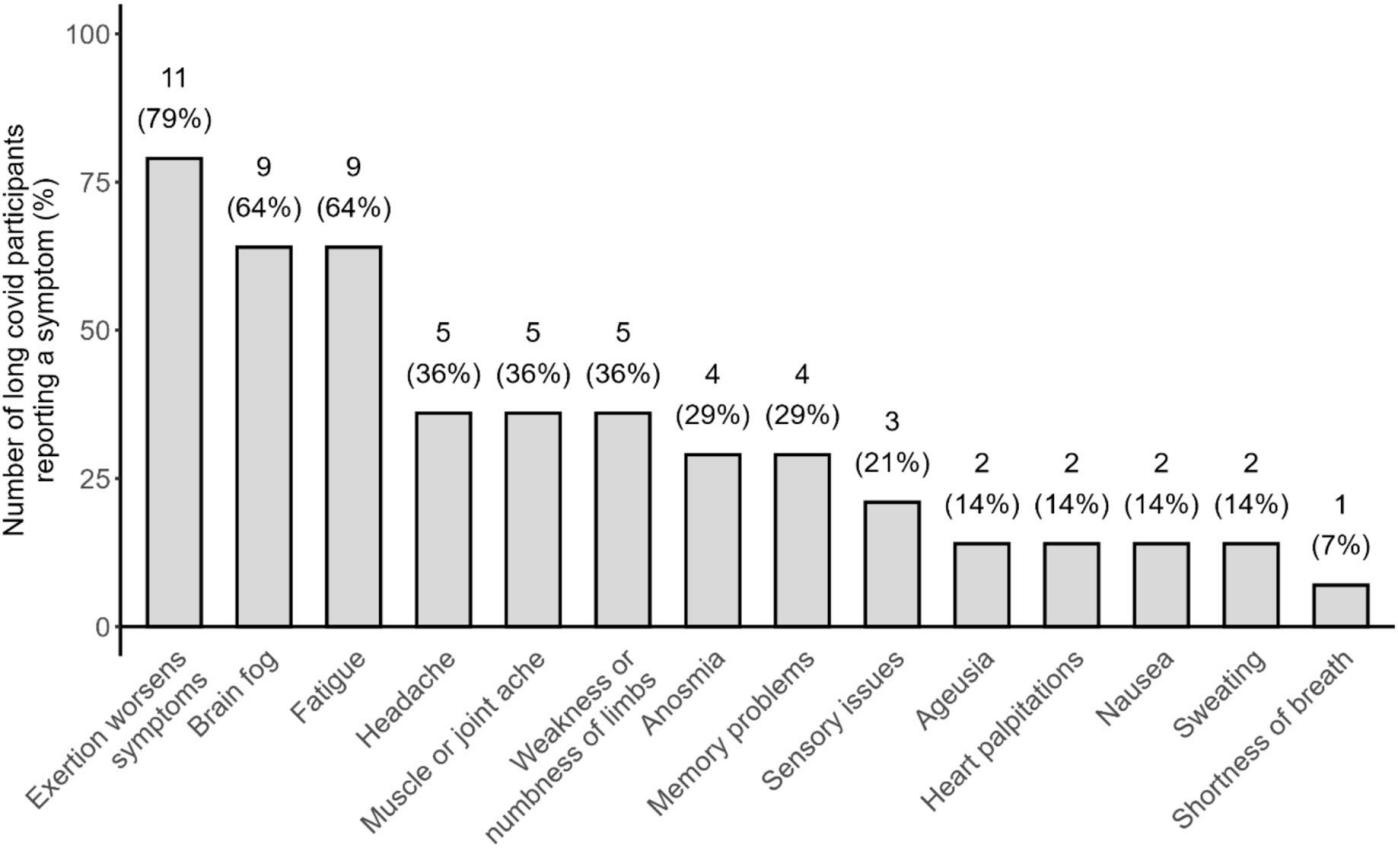
Symptoms reported by long COVID participants. The number of affected patients and symptom frequency is shown above the bar

LC duration from SARS-CoV-2 diagnosis ranged from 7 to 33 (median 15) months at the time of imaging. The most commonly reported symptom was post-exertional malaise, defined as the worsening of symptoms after physical or mental exertion, reported by 11 (79%) LC participants. Fatigue and “brain fog” were also common, reported by nine (64%) participants (Fig. 1).

All 14 LC participants underwent a clinical neurological examination. Seven participants (50%) showed a fully normal examination, while the remaining seven exhibited some neurological abnormalities. Four (29%) participants had impaired olfaction—two (14%) of whom also experienced loss of taste. Four (29%) had decreased sensation in the lower extremities (mainly impaired sense of vibration), and one (7%) had difficulties in toe-and-heel walking, jumping on one leg and squat sitting. Additionally, one participant had dysmetria (7%), and another presented a bilateral Babinski’s sign (7%).

The symptoms reported by the LC participants, apart from anosmia, were rarely accompanied by corresponding clinical examination abnormalities. For example, of the five participants reporting weakness or numbness of the limbs, three exhibited clinical symptoms upon the neurological examination: one reported impaired sensation, one had dysmetria, and one reported impaired olfaction. The remaining two had a fully normal neurological examination.

### Mental health questionnaire scores indicate elevated depression and anxiety in long COVID participants

EuroHIS-8, FSS, GAD-7, MFIS, ISI, PHQ-9, and WHODAS 2.0 questionnaire final scores are shown in Table 2. RAND-36 questionnaire scores by subsection, and subsection scores for the other questionnaires, are shown in Table S2. All questionnaire final scores were internally consistent at Cronbach’s α ≥ 0.70 and mean split-half reliability r ≥ 0.80.

**Table 2:** Mental health questionnaire scores among participants with long COVID

	Mean (SD)	Median (Q1–Q3)	Min–Max	Cronbach’s α	Split-half reliability mean *r*
Quality of life (EuroHIS-8)	24.3 (6.4)	21.0 (20.0–29.2)	17–34	0.90	0.89
Fatigue (FSS)	5.8 (1.4)	6.2 (5.8–6.6)	2–7	0.97	0.97
Fatigue (MFIS)	48.9 (20.2)	49.0 (40.5–61.5)	3–75	0.97	0.97
Sleep (ISI)	11.8 (5.3)	12.0 (7.5–13.8)	6–23	0.82	0.80
Anxiety (GAD-7)	12.5 (4.4)	12.5 (9.2–14.8)	7–20	0.90	0.89
Depression (PHQ-9)	8.4 (5.6)	6.5 (5.0–12.5)	2–17	0.88	0.87
Disability (WHODAS 2.0)	46.1 (23.5)	42.5 (38.8–60.0)	12–91	0.70	0.96

10 LC participants completed all questionnaires, one completed all but GAD-7 and ISI, and three did not complete any questionnaires. Means (SD) and medians (Q1–Q3) are calculated on questionnaire sum scores and Cronbach’s α across the corresponding individual questions. *FSS* Fatigue Severity Scale, *GAD-7* General Anxiety Disorder-7, *ISI* Insomnia Severity Index, *MFIS* Modified Fatigue Impact Scale, *PHQ-9* Patient Health Questionnaire 9, *WHODAS 2.0* WHO Disability Assessment Schedule 2.0

The LC participant PHQ-9 and GAD-7 mean scores were 8.4 and 12.5, indicating mild depression and moderate anxiety, respectively. Two participants demonstrated moderately severe depression with a PHQ-9 score of 17. Three participants had a GAD-7 score of 15 or higher, indicating severe anxiety.

Questionnaire scores did not correlate with duration of LC, weight, BMI or age, except for correlations between older age and higher fatigue questionnaire scores (FSS ρ = 0.68, *p* = 0.022 and MFIS ρ = 0.78, *p *= 0.005).

### TSPO binding does not differ between long COVID and healthy control participants

**Fig. 2:**
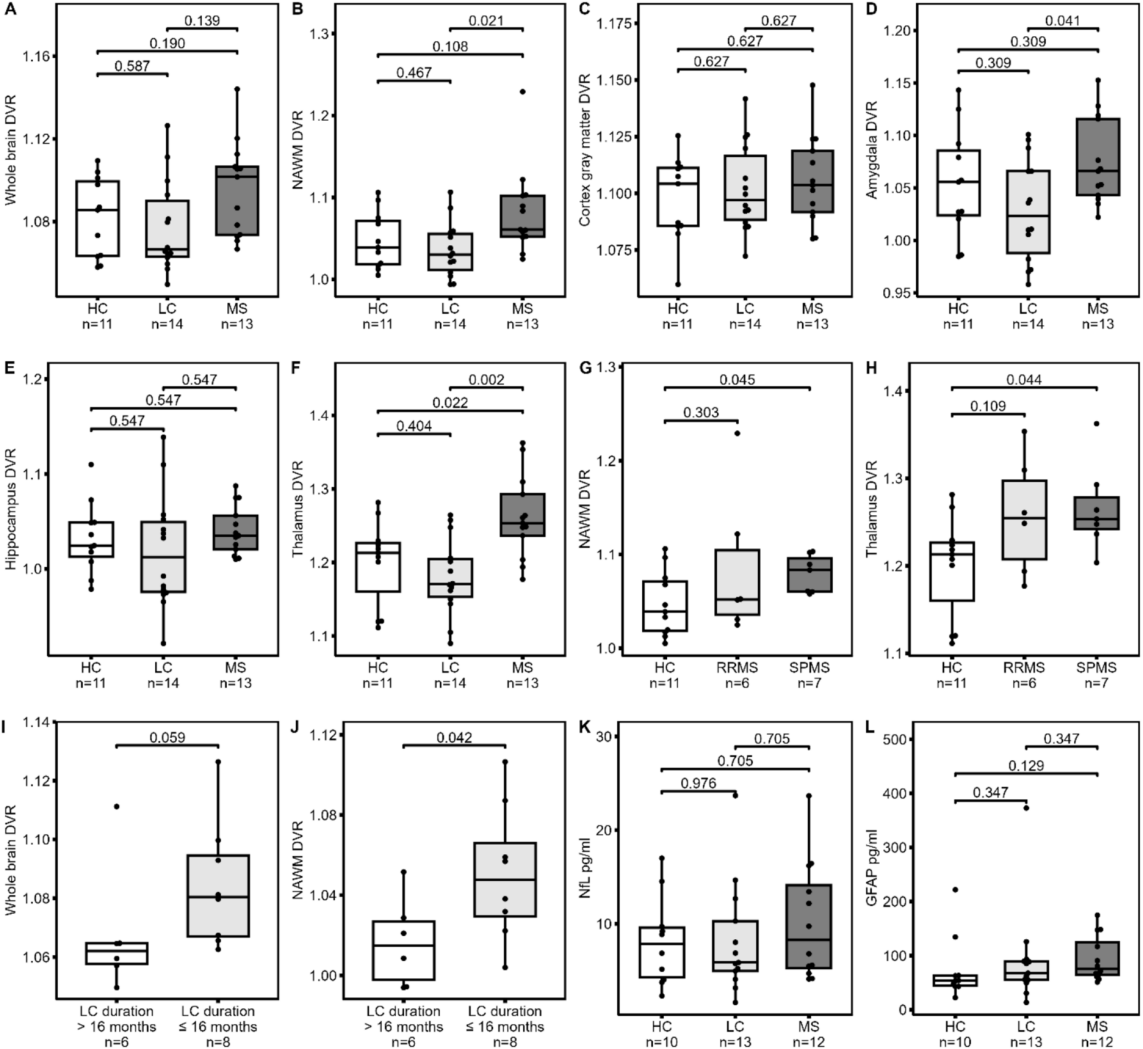
Regional [^11^C]PK11195 distribution volume ratios and serum biomarker levels in healthy controls, long COVID and multiple sclerosis participants. DVRs and within-parameter multiple-comparison-corrected (Benjamini–Hochberg method) *t*-test **A, C–H, J **or Wilcoxon test **B, I** *p*-values for group-wise comparisons are reported. **A**–**F** Whole brain, NAWM, cortical gray matter, amygdala, hippocampus and thalamus DVRs for all multiple sclerosis, LC and healthy control participants. **G**–**H** NAWM and thalamus DVRs for relapsing–remitting MS (RRMS), secondary progressive MS (SPMS) and healthy control participants. **I**–**J** Whole brain and NAWM DVRs in LC participants with SARS-CoV-2 diagnosis within 16 months of imaging versus over 16 months from imaging. **K**–**L** Serum concentration of GFAP and NfL biomarkers of astrocytic and axonal damage. GFAP and NfL were available in 13 LC participants, 10 healthy controls and 12 MS patients. *DVR* distribution volume ratio, *GFAP *glial fibrillary acidic protein, *HC* healthy control, *LC* long COVID, *MS* multiple sclerosis, *NAWM* normal-appearing white matter, *NfL *neurofilament light chain

There were no significant differences in [^11^C]PK11195 DVRs between LC and HC participants in any examined brain region (Table 3; Fig. 2A–F). Compared to MS patients, LC participants had lower DVRs across most brain regions with significant differences in the putamen, thalamus, amygdala and pallidum. Compared to HC participants, the MS group had higher DVRs in the thalamus (1.26 vs. 1.20, p < 0.001), and SPMS patients (Table S5) additionally in the NAWM (1.08 vs. 1.05, *p* = 0.023) (Fig. 2G–H, Table S6).

**Table 3:** Regional [^11^C]PK11195 distribution volume ratios in healthy control, long COVID and multiple sclerosis participants

	MS	HC	LC	HC vs MS	LC vs MS	HC vs LC
Whole brain DVR	1.10 (0.02)	1.08 (0.02)	1.08 (0.02)	0.126 (0.190)	**0.046** (0.139)	0.587 (0.587)
NAWM DVR	1.06 (1.05–1.10)	1.04 (1.02–1.07)	1.03 (1.01–1.06)	0.072 (0.108)	**0.007 (0.020)**	0.467 (0.467)
Cortex gray matter DVR	1.11 (0.02)	1.10 (0.02)	1.10 (0.02)	0.307 (0.627)	0.627 (0.627)	0.563 (0.627)
Amygdala DVR	1.07 (0.04)	1.05 (0.05)	1.03 (0.05)	0.309 (0.309)	**0.014 (0.041)**	0.220 (0.309)
Hippocampus DVR	1.04 (0.03)	1.03 (0.04)	1.02 (0.06)	0.547 (0.547)	0.219 (0.547)	0.480 (0.547)
Thalamus DVR	1.26 (0.06)	1.20 (0.06)	1.18 (0.05)	**0.015 (0.022)**	** < 0.001 (0.002)**	0.404 (0.404)
Putamen DVR	1.18 (0.06)	1.14 (0.04)	1.12 (0.04)	0.057 (0.085)	**0.006 (0.019)**	0.298 (0.298)
Pallidum DVR	1.18 (1.15–1.21)	1.16 (1.11–1.19)	1.11 (1.09–1.16)	0.258 (0.258)	**0.006 (0.017)**	0.149 (0.224)
Cingulate cortex DVR	1.09 (0.04)	1.09 (0.03)	1.09 (0.03)	0.548 (0.782)	0.782 (0.782)	0.700 (0.782)
Corpus callosum DVR	0.86 (0.85–0.92)	0.91 (0.88–0.91)	0.87 (0.84–0.89)	0.531 (0.796)	0.981 (0.981)	0.202 (0.607)
Brain stem DVR	1.23 (0.05)	1.20 (0.05)	1.18 (0.05)	0.076 (0.115)	**0.006 (0.018)**	0.326 (0.326)

For normally distributed parameters, mean (SD) and *t*-test p-values are reported; for non-normally distributed parameters, median (Q1–Q3) and Wilcoxon test *p*-values are reported. *P*-values in brackets are corrected for within-parameter multiple comparisons using the Benjamini–Hochberg method. Significant *p*-values are bolded. *DVR* distribution volume ratio, *HC* healthy control, *LC* long COVID, *MS* multiple sclerosis, *NAWM* normal-appearing white matter

LC participants imaged within 16 months of SARS-CoV-2 diagnosis (median LC duration 10 months, *n* = 8) had consistently higher DVRs in most regions compared to participants with longer time between diagnosis and imaging (median LC duration 26 months, *n* = 6) (Tables S7 and S8). The difference was significant for NAWM (1.05 vs. 1.02, *p* = 0.042) and trending to significance for the whole brain (1.08 vs. 1.06, *p* = 0.059) (Fig. 2I–J).

### MRI volumetric measurements do not differ between long COVID and healthy control participants

There were no significant differences between HC and LC participants in the volume parenchymal fractions of the examined brain regions (Table 4; Fig. S1A–F). The fractions were consistently higher in LC participants compared to MS patients, and the difference was significant in all brain areas except for the hippocampus and amygdala. MS parenchymal fractions of NAWM, thalamus, putamen, pallidum and brain stem were significantly lower than those in both LC and HC groups.

**Table 4:** Regional brain volume parenchymal fractions in healthy control, long COVID and multiple sclerosis participants

	MS	HC	LC	HC vs MS	LC vs MS	HC vs LC
Whole-brain volume PF	82.26 (3.85)	84.68 (3.11)	85.84 (2.25)	0.102 (0.153)	**0.009 (0.026)**	0.312 (0.312)
NAWM volume PF	32.39 (31.22–32.77)	35.00 (32.96–35.21)	34.75 (33.94–37.15)	**0.013 (0.019)**	** < 0.001 (0.001)**	0.291 (0.291)
Cortex gray matter volume PF	31.07 (2.06)	32.35 (2.23)	32.75 (1.54)	0.159 (0.239)	**0.025** (0.076)	0.618 (0.618)
Amygdala volume PF	0.24 (0.03)	0.24 (0.02)	0.24 (0.03)	0.736 (0.837)	0.648 (0.837)	0.837 (0.837)
Hippocampus volume PF	0.60 (0.07)	0.65 (0.04)	0.64 (0.06)	**0.022** (0.065)	0.068 (0.102)	0.731 (0.731)
Thalamus volume PF	0.93 (0.11)	1.09 (0.07)	1.10 (0.09)	** < 0.001 (0.001)**	** < 0.001 (< 0.001)**	0.949 (0.949)
Putamen volume PF	0.59 (0.07)	0.67 (0.07)	0.66 (0.06)	**0.007 (0.010)**	**0.007 (0.010)**	0.757 (0.757)
Pallidum volume PF	0.26 (0.24–0.27)	0.30 (0.27–0.30)	0.28 (0.27–0.30)	**0.005 (0.014)**	**0.043** (0.064)	0.501 (0.501)
Cingulate cortex volume PF	1.30 (0.12)	1.32 (0.19)	1.34 (0.13)	0.767 (0.767)	0.409 (0.767)	0.764 (0.767)
Corpus callosum volume PF	0.27 (0.04)	0.27 (0.03)	0.28 (0.02)	0.810 (0.810)	0.571 (0.810)	0.371 (0.810)
Brain stem volume PF	1.43 (0.18)	1.57 (0.06)	1.57 (0.12)	**0.020 (0.046)**	**0.030 (0.046)**	0.996 (0.996)

For normally distributed parameters, mean (SD) and *t*-test *p*-values are reported; for non-normally distributed parameters, median (Q1–Q3) and Wilcoxon test *p*-values are reported. *P*-values in brackets are corrected for within-parameter multiple comparisons using the Benjamini–Hochberg method. Significant *p*-values are bolded. *HC* healthy control, *LC* long COVID, *MS* multiple sclerosis, *NAWM* normal-appearing white matter, *PF* parenchymal fraction

### Serum biomarkers of neuronal and astrocytic damage do not differ between long COVID participants and healthy controls

NfL serum concentrations showed no significant differences between any of the study groups (Table 1; Fig. 2K–L). No significant differences were found in GFAP concentration between LC participants and HCs. The MS patients had higher GFAP concentration compared to HCs (median 76 vs. 68 pg/ml, *p* = 0.043), but the difference did not remain significant after multiple comparison correction (*p*= 0.13).

### TSPO binding correlates with mental health questionnaire scores in the long COVID cohort

**Fig. 3:**
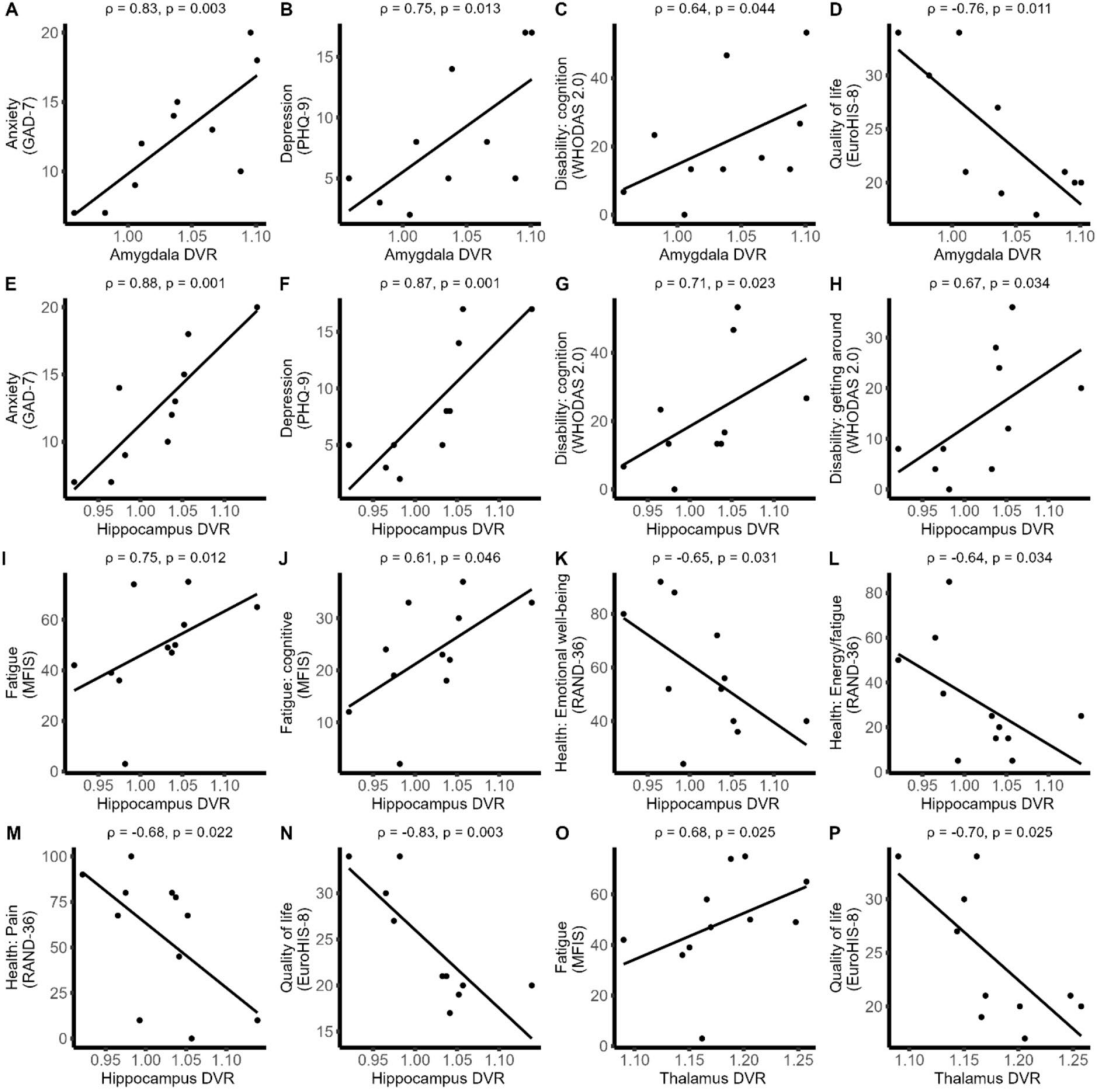
Significant Spearman correlations between mental health questionnaire scores and regional [^11^C]PK11195 distribution volume ratios in long COVID participants. **A**–**P** Correlations in amygdala, hippocampus and thalamus. Spearman correlation coefficients and associated raw *p*-values are shown along with linear regression-based trend lines. Questionnaire names in brackets. *FSS* Fatigue Severity Scale, *GAD-7* General Anxiety Disorder-7, *ISI* Insomnia Severity Index, *MFIS* Modified Fatigue Impact Scale, *NAWM* normal-appearing white matter, *PHQ-9* Patient Health Questionnaire-9, *RAND-36* RAND 36-Item Short Form Health Survey, *WHODAS 2.0* WHO Disability Assessment Schedule 2.0

**Fig. 4:**
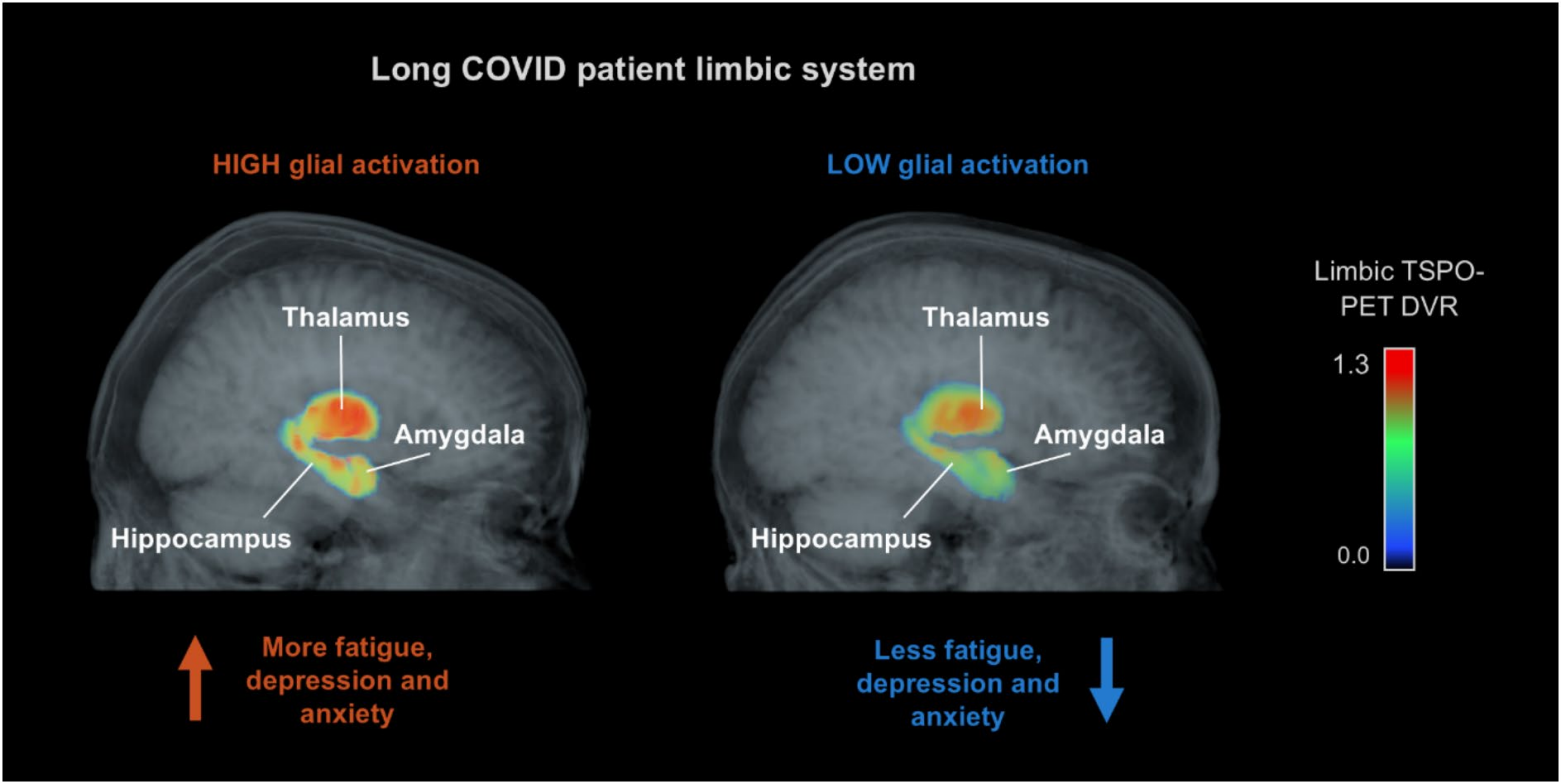
Fatigue, depression and anxiety correlate with limbic TSPO availability in LC participants. The figure shows glial activation in the limbic system of a female LC participant with high limbic TSPO availability (left) and a female LC participant with low limbic TSPO availability (right). Volumetric T1 MRI (grayscale) and limbic TSPO PET (colour gradient) images are overlaid and rendered with transparency to visualise TSPO availability throughout the limbic volume.

There were several significant correlations between LC participant questionnaire scores and DVRs in deep gray matter structures thalamus, hippocampus and amygdala (Table 5, Figs. 3A–P and 4). Lower EuroHIS-8 scores indicating decreased quality of life correlated strongly with higher DVRs in all three regions (ρ = − 0.70, p = 0.03; ρ = −0.83, p < 0.01; ρ = −0.76, p = 0.01). Higher GAD-7 and PHQ-9 scores evaluating anxiety and depression correlated strongly with increased hippocampus and amygdala DVRs (ρ = 0.88, *p*< 0.01; ρ = 0.75, *p* = 0.01).

**Table 5:** Spearman correlations between mental health questionnaire scores and regional [^11^C]PK11195 distribution volume ratios in long COVID participants

Questionnaire	Whole brain DVR	NAWM DVR	Cortex gray matter DVR	Amygdala DVR	Hippocampus DVR	Thalamus DVR
Quality of life (EuroHIS-8)	0.17 [-0.51–0.72] (0.64)	-0.09 [-0.68–0.57] (0.81)	0.43 [-0.27–0.84] (0.21)	-0.76 [-0.94–-0.25] **(0.01)**	-0.83 [-0.96–-0.42] **(< 0.01)**	-0.70 [-0.92–-0.12] **(0.03)**
Disability (WHODAS 2.0)	0.33 [-0.37–0.80] (0.35)	0.41 [-0.30–0.82] (0.24)	0.05 [-0.60–0.66] (0.88)	0.54 [-0.14–0.87] (0.11)	0.49 [-0.20–0.86] (0.15)	0.31 [-0.40–0.79] (0.38)
Fatigue (FSS)	0.21 [-0.44–0.72] (0.53)	0.17 [-0.48–0.70] (0.61)	0.13 [-0.51–0.68] (0.70)	0.11 [-0.52–0.67] (0.74)	0.35 [-0.31–0.79] (0.29)	0.32 [-0.35–0.77] (0.35)
Fatigue (MFIS)	0.30 [-0.37–0.76] (0.37)	0.37 [-0.29–0.79] (0.26)	-0.03 [-0.62–0.58] (0.94)	0.50 [-0.14–0.85] (0.12)	0.75 [0.26–0.93] **(0.01)**	0.68 [0.14–0.91] **(0.02)**
Anxiety (GAD-7)	0.05 [-0.60–0.66] (0.89)	0.36 [-0.35–0.81] (0.31)	-0.26 [-0.76–0.45] (0.48)	0.83 [0.41–0.96] **(< 0.01)**	0.88 [0.55–0.97] **(< 0.01)**	0.57 [-0.10–0.88] (0.09)
Depression (PHQ-9)	0.07 [-0.59–0.67] (0.85)	0.22 [-0.48–0.74] (0.55)	-0.29 [-0.78–0.42] (0.42)	0.75 [0.22–0.94] **(0.01)**	0.87 [0.53–0.97] **(< 0.01)**	0.59 [-0.06–0.89] (0.07)
Sleep (ISI)	0.01 [-0.63–0.63] (0.99)	0.23 [-0.47–0.75] (0.52)	-0.26 [-0.77–0.44] (0.46)	0.49 [-0.20–0.85] (0.15)	0.59 [-0.07–0.89] (0.08)	0.27 [-0.43–0.77] (0.44)

Spearman correlation coefficients, 5% to 95% confidence intervals [0.05–0.95 CI] and naive *t*-test *p*-values (*p*) are shown. Significant *p*-values are bolded. *FSS* Fatigue Severity Scale, *GAD-7* General Anxiety Disorder-7, *ISI* Insomnia Severity Index, *MFIS* Modified Fatigue Impact Scale, *NAWM* normal-appearing white matter, *PHQ-9* Patient Health Questionnaire-9, *WHODAS 2.0* WHO Disability Assessment Schedule 2.0

Of the fatigue questionnaires, an increased MFIS score, which includes questions about mental fatigue, correlated with higher DVRs in the hippocampus and thalamus. In contrast, the FSS questionnaire, in which mental and physical fatigue are not evaluated separately, did not correlate with any DVRs.

Overall health and disability questionnaire WHODAS 2.0 showed no correlation with DVRs in any region, but the cognitive section subscore correlated with increased hippocampal (ρ = 0.71, *p*= 0.02) and amygdala (ρ = 0.64, *p* = 0.04) DVRs (Tables S3, S4, Fig. 3C, G). Similarly, worse RAND-36 energy/fatigue-, pain- and emotional wellbeing subsection scores, but not general health subsection scores, correlated with higher DVRs in the hippocampus (Table S3, Fig. 3).

The sleep evaluation questionnaire ISI showed no significant correlation with DVRs in any studied region.

Across all comparisons, 16 of the 17 identified significant correlations were in the limbic system. A permutation enrichment analysis was used to assess whether this concentration exceeds the number expected by chance. The analysis was restricted to the questionnaire scores that were independent of each other by excluding questionnaire subsection scores. Of the 13 remaining correlations, 12 concentrated in the anatomically predefined limbic system, representing a more than threefold enrichment (permutation p = 0.006), inconsistent with a multiple-testing artefact. The one significant correlation outside the limbic system was between putamen DVR and RAND social functioning (ρ = − 0.70, *p* = 0.02) (Table S4).

## Discussion

To our knowledge, this is the first study to employ both positive and negative control groups to investigate neuroinflammation in a LC cohort with neurological and psychiatric symptoms. We found no significant differences in [^11^C]PK11195 DVR, a measure of TSPO availability associated with glial cell activation, in any brain region between LC and HC participants. LC participants consistently demonstrated lower DVRs, indicative of lower TSPO availability, than the positive neuroinflammation control group of MS patients. Compared to HCs, MS patients showed higher TSPO availability in the thalamus [30], while SPMS patients additionally showed higher expression in the NAWM [31], validating the imaging methodology.

While we did not observe altered regional TSPO availability in LC participants compared to the HCs, we found strong positive associations between LC mental health symptom severity and [^11^C]PK11195 DVRs in limbic regions—including the hippocampus, amygdala, and thalamus. This regionally selective association in the absence of elevated global glial activation suggests that the increased TSPO availability in the limbic areas may reflect the functional central nervous system (CNS) alterations associated with the neurological and psychiatric LC symptoms rather than residual elevated inflammation from the acute SARS-Cov-2 infection. It is important to note that TSPO availability is relatively unspecific, and aside from inflammation, also reflects other cellular functions such as mitochondrial metabolic activity and the density of TSPO-expressing glial cells actively performing functions not related to inflammation (reviewed in [32]). Thus, TSPO availability may reflect CNS activation in LC without necessarily indicating ongoing proinflammatory processes [33, 34]. Glial cells contribute to several essential brain functions—including debris clearance, synaptic pruning, and neuromodulation (reviewed in [35])—which may be particularly active in the limbic system in chronic neuropsychiatric conditions.

Indeed, similarities in clinical presentation and neuroimaging findings have been reported across LC, myalgic encephalomyelitis/chronic fatigue syndrome (ME/CFS), and depression [36, 37]. Nakatomi et al. (2014), for example, reported increased TSPO expression in the hippocampus, amygdala, and thalamus in ME/CFS patients with neuropsychiatric symptoms [38]. Meta-analyses of TSPO PET imaging in depression have likewise shown increased expression in limbic structures, including the anterior cingulate cortex (ACC) and hippocampus [39, 40]. Our findings align with these reports, suggesting that increased TSPO availability in limbic regions may associate with a shared neurobiological mechanism underlying the chronic psychiatric and neurological symptoms. Similar “sickness behaviour” symptoms—such as fatigue, sleep disturbances, anhedonia, and cognitive impairment—across LC, ME/CFS, and depression further support this interpretation.

In line with the lack of LC-associated elevated neuroinflammation in our study cohort, Visser et al. (2025) reported no difference in TSPO availability between 33 individuals with persistent post-COVID-19 symptoms and 14 individuals without post-infection symptoms using [^18^F]DPA-714 radioligand [41]. However, earlier studies have reported increased TSPO expression in LC. VanElzakker et al. (2024) reported elevated [^11^C]PBR28 radioligand standardised uptake value (SUVR) in the limbic system, anterior cingulate cortex, thalamus, and basal ganglia in LC patients compared to pre-pandemic controls [9]. Braga et al. (2023) found increased [^18^F]FEPPA radioligand binding in the ventral striatum and dorsal putamen in moderately depressed LC patients, though without correlation to symptom severity or cognitive function [10].

One potential explanation for these discrepancies is the use of different radioligands. While our study used the first-generation tracer [^11^C]PK11195, others have employed second-generation tracers [^11^C]PBR28 and [^18^F]FEPPA. Although [^11^C]PK11195 is widely used and has well-established analysis methods, it has a lower signal-to-noise ratio due to high nonspecific binding, which may reduce its sensitivity to subtle inflammatory changes [42, 43]. This limitation could explain why our study did not detect elevated TSPO availability in LC, while still capturing the pronounced increases observed in MS patients.

Notably, however, the earlier PET studies reporting elevated neuroinflammation have focussed on participants imaged within 6 months of infection [10], or selected those with severe ME/CFS-like symptom profiles [9], which may account for the higher observed inflammation [14]. In contrast, our inclusion criterion resulted in a cohort with mean disease duration of 17 months and, similar to the Visser et al. 2025 cohort with 25-month mean disease duration, reflected the effects of established chronic disease rather than acute post-infection changes. Consistently, we observed higher NAWM DVRs in participants with more recent infections, suggesting that neuroinflammation may be more prominent early on, potentially resolving over time while symptoms persist. This may suggest a shift from neuroinflammatory to other mechanisms, such as increased neuronal activity and the concomitant alteration of glial metabolism in the limbic areas, as contributors to LC symptomatology [36].

Regarding peripheral biomarkers, we found no differences in GFAP or NfL levels between the LC and HC groups. Although both markers are elevated during the acute phase of COVID-19 [12], our finding is consistent with previous studies reporting lack of post-infection NfL elevation [15, 17]. Furthermore, elevated GFAP levels measured at an early post-infection stage return to normal levels by the 6-month post-infection timepoint [14–17].

## Limitations

This study included a relatively small sample of 14 LC patients, 11 HCs, and 13 MS patients. Future research should recruit larger LC cohorts, either targeting participants with well-defined symptom subtypes, duration and severity, or defining the population more broadly while incorporating precise clinical stratifications that capture greater phenotypic diversity of LC symptoms and durations.

Furthermore, while group differences in age and sex were not significant, LC participants had a nearly significantly higher BMI compared to HCs, which may represent a potential confounder. However, correcting TSPO availability for BMI differences did not diminish the association of neuropsychiatric symptoms with limbic TSPO availability. Five HC participants had prior COVID-19 diagnoses. It would have been more attractive to have an entirely COVID-naive HC cohort, but the high population-level exposure to SARS-CoV-2 prior to and during the study period means that truly COVID-naive controls were not practically attainable. Moreover, the absence of a documented diagnosis does not preclude prior undetected infection. Nonetheless, the HCs with and without prior COVID diagnosis were devoid of any LC symptoms, and did not differ in the measured inflammatory parameters. Therefore, the risk that a previous exposure to SARS-CoV-2 among the healthy controls would have confounded the findings can be considered negligible.

## Conclusion

We found no evidence of increased TSPO availability or elevated serum biomarkers indicative of CNS damage in LC patients experiencing psychiatric and cognitive symptoms on average 17 months post-infection. Moreover, NAWM TSPO availability was lower in participants with a prolonged LC duration compared to those with more recent SARS-CoV-2 infection. These findings suggest that persistent LC symptoms are unlikely to be driven by ongoing elevated neuroinflammation. However, LC symptom severity correlated positively with TSPO availability in the limbic regions. This may imply that residual COVID-19-associated elevated neuroinflammation observed in early LC subsides with time and does not explain the persistent LC symptoms which may instead reflect cellular metabolic changes in mood- and cognition-related brain areas.

## Supplementary Information

Below is the link to the electronic supplementary material.

Supplementary file1 (DOCX 139 KB)

## Data Availability

Data are available on reasonable request. Anonymised data not published within the article will be shared on a request from a qualified investigator.

## References

[1] World Health Organization. A clinical case definition of post COVID-19 condition by a Delphi consensus, 6 October 2021. document number: WHO/2019-nCoV/Post_COVID-19_condition/Clinical_case_definition/20211. Published Online First: 2021.

[2] Davis HE, Assaf GS, McCorkell L, et al. Characterizing long COVID in an international cohort: 7 months of symptoms and their impact. eClinicalMedicine. 2021;38:101019. 10.1016/j.eclinm.2021.101019 10.1016/j.eclinm.2021.101019 PMC8280690 34308300

[3] Zeng N, Zhao Y-M, Yan W et al (2023) A systematic review and meta-analysis of long term physical and mental sequelae of COVID-19 pandemic: call for research priority and action. Mol Psychiatry 28:423–433. 10.1038/s41380-022-01614-7 35668159 10.1038/s41380-022-01614-7 PMC9168643

[4] Altmann DM, Whettlock EM, Liu S et al (2023) The immunology of long COVID. Nat Rev Immunol 23:618–634. 10.1038/s41577-023-00904-7 37433988 10.1038/s41577-023-00904-7

[5] López LC (2024) COVID’s toll on the brain: new clues emerge. Nature 628:20–20. 10.1038/d41586-024-00828-9 38509295 10.1038/d41586-024-00828-9

[6] Monje M, Iwasaki A (2022) The neurobiology of long COVID. Neuron 110:3484–3496. 10.1016/j.neuron.2022.10.006 36288726 10.1016/j.neuron.2022.10.006 PMC9537254

[7] Davis HE, McCorkell L, Vogel JM et al (2023) Long COVID: major findings, mechanisms and recommendations. Nat Rev Microbiol 21:133–146. 10.1038/s41579-022-00846-2 36639608 10.1038/s41579-022-00846-2 PMC9839201

[8] Turkheimer FE, Rizzo G, Bloomfield PS et al (2015) The methodology of TSPO imaging with positron emission tomography. Biochem Soc Trans 43:586–592. 10.1042/BST20150058 26551697 10.1042/BST20150058 PMC4613512

[9] VanElzakker MB, Bues HF, Brusaferri L et al (2024) Neuroinflammation in post-acute sequelae of COVID-19 (PASC) as assessed by [11C]PBR28 PET correlates with vascular disease measures. Brain Behav Immun 119:713–723. 10.1016/j.bbi.2024.04.015 38642615 10.1016/j.bbi.2024.04.015 PMC11225883

[10] Braga J, Lepra M, Kish SJ et al (2023) Neuroinflammation After COVID-19 With Persistent Depressive and Cognitive Symptoms. JAMA Psychiat 80:787–795. 10.1001/jamapsychiatry.2023.1321 10.1001/jamapsychiatry.2023.1321 PMC10233457 37256580

[11] Filippo MD, Gaetani L, Centonze D, et al. Fluid biomarkers in multiple sclerosis: from current to future applications. The Lancet Regional Health – Europe. 2024; 10.1016/j.lanepe.2024.101009 10.1016/j.lanepe.2024.101009 PMC11496979 39444698

[12] Pilotto A, Masciocchi S, Volonghi I et al (2021) Severe Acute Respiratory Syndrome Coronavirus 2 (SARS-CoV-2) Encephalitis Is a Cytokine Release Syndrome: Evidences From Cerebrospinal Fluid Analyses. Clin Infect Dis 73:e3019–e3026. 10.1093/cid/ciaa1933 33395482 10.1093/cid/ciaa1933 PMC7799260

[13] Zingaropoli MA, Pasculli P, Barbato C et al (2023) Biomarkers of Neurological Damage: From Acute Stage to Post-Acute Sequelae of COVID-19. Cells 12:2270. 10.3390/cells12182270 37759493 10.3390/cells12182270 PMC10526816

[14] Kanberg N, Simrén J, Edén A, et al. Neurochemical signs of astrocytic and neuronal injury in acute COVID-19 normalizes during long-term follow-up. eBioMedicine. 2021;70:103512. 10.1016/j.ebiom.2021.103512 10.1016/j.ebiom.2021.103512 PMC8320425 34333238

[15] Lennol MP, Ashton NJ, Moreno-Pérez O et al (2023) Transient changes in the plasma of astrocytic and neuronal injury biomarkers in COVID-19 patients without neurological syndromes. Int J Mol Sci 24:2715. 10.3390/ijms24032715 36769057 10.3390/ijms24032715 PMC9917569

[16] Vrettou CS, Vassiliou AG, Keskinidou C et al (2024) A prospective study on neural biomarkers in patients with long-COVID symptoms. J Pers Med 14:313. 10.3390/jpm14030313 38541055 10.3390/jpm14030313 PMC10971257

[17] Peluso MJ, Sans HM, Forman CA et al (2022) Plasma markers of neurologic injury and inflammation in people with self-reported neurologic postacute sequelae of SARS-CoV-2 infection. Neurol Neuroimmunol Neuroinflamm 9:e200003. 10.1212/NXI.0000000000200003 35701186 10.1212/NXI.0000000000200003 PMC9210548

[18] Nosikov A, Gudex C (2003) EUROHIS: Developing common instruments for health surveys. Published on behalf of the World Health Organization Regional Office for Europe by IOS Press ; Ohmsha, Amsterdam, Washington, D.C.

[19] Hays RD, Morales LS (2001) The RAND-36 measure of health-related quality of life. Ann Med 33:350–357. 10.3109/07853890109002089 11491194 10.3109/07853890109002089

[20] Krupp LB, LaRocca NG, Muir-Nash J (1989) The fatigue severity scale: application to patients with multiple sclerosis and systemic lupus erythematosus. Arch Neurol 46:1121–1123. 10.1001/archneur.1989.00520460115022 2803071 10.1001/archneur.1989.00520460115022

[21] Fisk JD, Ritvo PG, Ross L et al (1994) Measuring the functional impact of fatigue: initial validation of the fatigue impact scale. Clin Infect Dis 18:S79-83. 10.1093/clinids/18.Supplement_1.S79 8148458 10.1093/clinids/18.supplement_1.s79

[22] Spitzer RL, Kroenke K, Williams JBW et al (2006) A brief measure for assessing generalized anxiety disorder: the GAD-7. Arch Intern Med 166:1092–1097. 10.1001/archinte.166.10.1092 16717171 10.1001/archinte.166.10.1092

[23] Kroenke K, Spitzer RL, Williams JBW (2001) The PHQ-9. J Gen Intern Med 16:606–613. 10.1046/j.1525-1497.2001.016009606.x 11556941 10.1046/j.1525-1497.2001.016009606.x PMC1495268

[24] Morin CM, Belleville G, Bélanger L et al (2011) The insomnia severity index: psychometric indicators to detect insomnia cases and evaluate treatment response. Sleep 34:601–608. 10.1093/sleep/34.5.601 21532953 10.1093/sleep/34.5.601 PMC3079939

[25] Rehm J, Üstün TB, Saxena S et al (1999) On the development and psychometric testing of the WHO screening instrument to assess disablement in the general population. Int J Methods Psychiatr Res 8:110–122. 10.1002/mpr.61

[26] Saraste M, Matilainen M, Vuorimaa A et al (2023) Association of serum neurofilament light with microglial activation in multiple sclerosis. J Neurol Neurosurg Psychiatry 94:698–706. 10.1136/jnnp-2023-331051 37130728 10.1136/jnnp-2023-331051 PMC10447382

[27] Rissanen E, Tuisku J, Vahlberg T et al (2018) Microglial activation, white matter tract damage, and disability in MS. Neurol Neuroimmunol Neuroinflamm 5:e443. 10.1212/NXI.0000000000000443 29520366 10.1212/NXI.0000000000000443 PMC5840890

[28] Turkheimer FE, Edison P, Pavese N et al (2007) Reference and Target Region Modeling of [11C]-(R)-PK11195 Brain Studies. J Nucl Med 48:158–167 17204713

[29] Yaqub M, van Berckel BN, Schuitemaker A et al (2012) Optimization of supervised cluster analysis for extracting reference tissue input curves in (R)-[11C]PK11195 brain PET studies. J Cereb Blood Flow Metab 32:1600–1608. 10.1038/jcbfm.2012.59 22588187 10.1038/jcbfm.2012.59 PMC3421099

[30] Misin O, Matilainen M, Nylund M et al (2022) Innate immune cell–related pathology in the thalamus signals a risk for disability progression in multiple sclerosis. Neurol Neuroimmunol Neuroinflamm 9:e1182. 10.1212/NXI.0000000000001182 35581004 10.1212/NXI.0000000000001182 PMC9128041

[31] Nylund M, Sucksdorff M, Matilainen M, *et al.* Phenotyping of multiple sclerosis lesions according to innate immune cell activation using 18 kDa translocator protein-PET. *Brain Commun*. 2021;4:fcab301. 10.1093/braincomms/fcab301 10.1093/braincomms/fcab301 PMC8727984 34993478

[32] Cheung G, Lin YC, Papadopoulos V. Translocator protein in the rise and fall of central nervous system neurons. Front Cell Neurosci. 2023;. 10.3389/fncel.2023.1210205 10.3389/fncel.2023.1210205 PMC10322222 37416505

[33] Notter T, Schalbetter SM, Clifton NE et al (2021) Neuronal activity increases translocator protein (TSPO) levels. Mol Psychiatry 26:2025–2037. 10.1038/s41380-020-0745-1 32398717 10.1038/s41380-020-0745-1 PMC8440208

[34] Estes ML, McAllister AK (2014) Alterations in immune cells and mediators in the brain: it’s not always neuroinflammation! Brain Pathol 24:623–630. 10.1111/bpa.12198 25345893 10.1111/bpa.12198 PMC4365495

[35] Allen NJ, Lyons DA (2018) Glia as architects of central nervous system formation and function. Science 362:181–185. 10.1126/science.aat0473 30309945 10.1126/science.aat0473 PMC6292669

[36] Komaroff AL, Lipkin WI (2023) ME/CFS and long COVID share similar symptoms and biological abnormalities: road map to the literature. Front Med 10:1187163. 10.3389/fmed.2023.1187163 10.3389/fmed.2023.1187163 PMC10278546 37342500

[37] Tate W, Walker M, Sweetman E et al (2022) Molecular mechanisms of neuroinflammation in ME/CFS and long COVID to sustain disease and promote relapses. Front Neurol 13:877772. 10.3389/fneur.2022.877772 35693009 10.3389/fneur.2022.877772 PMC9174654

[38] Nakatomi Y, Mizuno K, Ishii A et al (2014) Neuroinflammation in patients with Chronic Fatigue Syndrome/Myalgic Encephalomyelitis: an 11C-(R)-PK11195 PET study. J Nucl Med 55:945–950. 10.2967/jnumed.113.131045 24665088 10.2967/jnumed.113.131045

[39] Enache D, Pariante CM, Mondelli V (2019) Markers of central inflammation in major depressive disorder: a systematic review and meta-analysis of studies examining cerebrospinal fluid, positron emission tomography and post-mortem brain tissue. Brain Behav Immun 81:24–40. 10.1016/j.bbi.2019.06.015 31195092 10.1016/j.bbi.2019.06.015

[40] Eggerstorfer B, Kim J-H, Cumming P et al (2022) Meta-analysis of molecular imaging of translocator protein in major depression. Front Mol Neurosci. 10.3389/fnmol.2022.981442 36226319 10.3389/fnmol.2022.981442 PMC9549359

[41] Visser D, Golla SSV, Palard-Novello X et al (2025) Varying levels of inflammatory activity in brain and body of patients with persistent fatigue and difficulty concentrating after COVID-19: a TSPO PET study. J Nucl Med 66:1787–1794. 10.2967/jnumed.124.269297 40935606 10.2967/jnumed.124.269297 PMC12582180

[42] Chauveau F, Becker G, Boutin H (2021) Have (R)-[11C]PK11195 challengers fulfilled the promise? A scoping review of clinical TSPO PET studies. Eur J Nucl Med Mol Imaging 49:201–220. 10.1007/s00259-021-05425-w 34387719 10.1007/s00259-021-05425-w PMC8712292

[43] Zhang L, Hu K, Shao T et al (2021) Recent developments on PET radiotracers for TSPO and their applications in neuroimaging. Acta Pharm Sin B 11:373–393. 10.1016/j.apsb.2020.08.006 33643818 10.1016/j.apsb.2020.08.006 PMC7893127

