# Supplementary material for: Association between post-COVID-19 neuropsychiatric symptoms and persistent glial activation in the limbic system: a TSPO PET study
Source: J Neurol. 2026 Apr 30;273(5):298. doi: 10.1007/s00415-026-13842-w (PMC13132884; doi:10.1007/s00415-026-13842-w)
Supplement: Supplementary file 1 — Supplementary file1 (DOCX 139 KB) [file 415_2026_13842_MOESM1_ESM.docx]

**Journal of Neurology**

**Association Between Post-COVID-19 Neuropsychiatric Symptoms and Persistent Glial Activation in the Limbic System: A TSPO PET study**

Authors: Joel Tuomaala^1,2,3,4^, Maija Saraste^1,2,3,4^, Emma Smith^3^, Matilda Kuusi^1^, Elisabet Westerberg^5^, Eveliina Honkonen^1,2,3,4^, Rahim Kargar^2^, Sini Laaksonen^1,2,3^, Jussi Lehto^1,2,4^, Amelie Luoma^1,3^, Markus Matilainen^1,2,3,4^ Olavi Misin^1,2,3^, Janne Atosuo^6^, Mari Kanerva^7^, Helena Liira^8^, Sini Laakso^8^, Tatiana Posharina^8^, Virva Saunavaara^1^, Saara Wahlroos^9^, Johan Rajander^10^, Laura Airas^1,2,3,4^

^1^ Turku PET Centre, Turku University Hospital, University of Turku, and Åbo Akademi University, Turku, Finland

^2^ Neurocenter, Turku University Hospital, Turku, Finland

^3^ Clinical Neurosciences, University of Turku, Turku, Finland

^4^ InFLAMES Research Flagship, University of Turku, Turku, Finland

^5^ Åland Central Hospital, Mariehamn, Åland, Finland

^6^ Department of Biotechnology, University of Turku, Turku, Finland

^7^ Department of Hospital Hygiene and Infection Control, TYKS Turku University Hospital, Turku, Finland

^8^Outpatient Clinic for Long-Term Effects of COVID-19, Helsinki University Central Hospital, and University of Helsinki, Helsinki, Finland

^9^ Radiopharmaceutical Chemistry Laboratory, Turku PET Centre, University of Turku

^10^ Accelerator Laboratory, Turku PET Centre, Åbo Akademi University, Turku, Finland

ES, MS, JT contributed equally to this work

**Correspondence to** Prof Laura Airas MD, PhD, Itäinen Pitkäkatu 4 A, Turku PET Centre, Turku University Hospital, University of Turku, and Åbo Akademi University, Turku, Finland; [laura.airas@utu.fi](mailto:laura.airas@utu.fi)

# SUPPLEMENTARY MATERIAL

**Table S1. Demographic, soluble biomarker, and DVRs of healthy control participants with past SARS-CoV-2 diagnosis and without past SARS-CoV-2 diagnosis**

|  | **HC (past COVID)** | **HC (no COVID)** | **HC (past COVID) vs. HC (no COVID)** |
| --- | --- | --- | --- |
| n | 6 | 5 |  |
| n_m_f | 4/2 | 3/2 | 1.000 |
| Age | 48.33 (13.02) | 42.20 (11.50) | 0.428 |
| BMI | 27.19 (24.39 - 30.87) | 26.24 (25.76 - 27.16) | 0.429 |
| NfL | 9.57 (6.18) | 6.71 (2.80) | 0.385 |
| GFAP | 105.17 (73.96) | 44.14 (12.97) | 0.139 |
| NAWM DVR | 1.05 (0.05) | 1.04 (0.02) | 0.606 |
| Cortex gray matter DVR | 1.10 (0.01) | 1.10 (0.03) | 0.827 |
| Brain stem DVR | 1.21 (0.03) | 1.19 (0.06) | 0.697 |
| Cingulate cortex DVR | 1.09 (0.03) | 1.08 (0.04) | 0.840 |
| Thalamus DVR | 1.21 (0.05) | 1.19 (0.07) | 0.721 |
| Hippocampus DVR | 1.03 (0.03) | 1.04 (0.05) | 0.821 |
| Putamen DVR | 1.15 (0.04) | 1.13 (0.05) | 0.586 |
| Pallidum DVR | 1.16 (0.04) | 1.15 (0.07) | 0.939 |
| Amygdala DVR | 1.06 (0.04) | 1.05 (0.07) | 0.918 |
| Caudate DVR | 0.91 (0.03) | 0.92 (0.02) | 0.561 |
| Whole brain DVR | 1.08 (0.02) | 1.08 (0.02) | 0.824 |

For normally distributed parameters, mean (SD) and t-test p-values are reported; for non-normally distributed parameters median (Q1-Q3) and Wilcoxon test p-values are reported. BMI, body weight index; DVR, distribution volume ratio; HC, healthy control; NfL, neurofilament light chain; GFAP, glial fibrillary acidic protein.

**Table S2: Final and sub section questionnaire scores in long covid participants**

| **Questionnaire** | **Mean (SD)** | **Median (Q1 - Q3)** | **Min - max** | **Cronbach's α** | **Split-half reliability mean r** |
| --- | --- | --- | --- | --- | --- |
| **Quality of life (EuroHIS-8)** | 24.3 (6.4) | 21.0 (20.0 - 29.2) | 17 - 34 | 0.90 | 0.89 |
| **Disability (WHODAS 2.0)** | 46.1 (23.5) | 42.5 (38.8 - 60.0) | 12 - 91 | 0.70 | 0.96 |
| **Disability: cognition (WHODAS 2.0)** | 21.3 (16.9) | 15.0 (13.3 - 25.8) | 0 – 53.3 | 0.88 | 0.89 |
| **Disability: social (WHODAS 2.0)** | 32.5 (16.9) | 30.0 (20.0 - 45.0) | 10 - 60 | 0.68 | 0.74 |
| **Disability: household activities (WHODAS 2.0)** | 28.0 (19.5) | 30.0 (10.0 - 42.5) | 0 - 55 | 0.95 | 0.96 |
| **Disability: work activities (WHODAS 2.0)** | 52.0 (31.9) | 67.5 (25.0 - 80.0) | 0 - 80 | 0.99 | 0.99 |
| **Disability: societal (WHODAS 2.0)** | 32.2 (16.9) | 33.8 (24.4 - 41.2) | 0 - 60 | 0.87 | 0.86 |
| **Disability: self-care (WHODAS 2.0)** | 3.5 (6.7) | 0.0 (0.0 - 3.8) | 0 - 20 | 0.86 | 0.87 |
| **Disability: getting around (WHODAS 2.0)** | 14.4 (12.0) | 10.0 (5.0 - 23.0) | 0 - 36 | 0.85 | 0.83 |
| **Fatigue (FSS)** | 5.8 (1.4) | 6.2 (5.8 - 6.6) | 2 - 7 | 0.97 | 0.96 |
| **Fatigue (MFIS)** | 48.9 (20.2) | 49.0 (40.5 - 61.5) | 3 - 75 | 0.97 | 0.97 |
| **Fatigue: physical (MFIS)** | 21.1 (9.3) | 24.0 (17.0 - 24.5) | 1 - 33 | 0.93 | 0.91 |
| **Fatigue: cognitive (MFIS)** | 23.0 (10.2) | 23.0 (18.5 - 31.5) | 2 - 37 | 0.96 | 0.96 |
| **Fatigue: psychosocial (MFIS)** | 4.8 (2.3) | 5.0 (4.0 - 6.0) | 0 - 8 | 0.79 | 0.80 |
| **Anxiety (GAD-7)** | 12.5 (4.4) | 12.5 (9.2 - 14.8) | 7 - 20 | 0.90 | 0.89 |
| **Depression (PHQ-9)** | 8.4 (5.6) | 6.5 (5.0 - 12.5) | 2 - 17 | 0.88 | 0.87 |
| **Sleep (ISI)** | 11.8 (5.3) | 12.0 (7.5 - 13.8) | 6 - 23 | 0.82 | 0.80 |
| **Health: General health (RAND-36)** | 39.1 (22.9) | 35.0 (27.5 - 42.5) | 10 - 85 | 0.97 | 0.97 |
| **Health: Energy/fatigue (RAND-36)** | 30.9 (24.9) | 25.0 (15.0 - 42.5) | 5 – 85 | 0.90 | 0.90 |
| **Health: Emotional well-being (RAND-36)** | 57.5 (22.6) | 52.0 (40.0 - 76.0) | 24 - 92 | 0.93 | 0.90 |
| **Health: Physical functioning (RAND-36)** | 61.4 (22.8) | 55.0 (50.0 - 77.5) | 25 - 100 | 0.90 | 0.91 |
| **Health: Social functioning (RAND-36)** | 42.0 (28.7) | 37.5 (18.8 - 56.2) | 12.5 - 100 | 0.66 | 0.66 |
| **Health: Pain (RAND-36)** | 57.0 (35.3) | 67.5 (27.5 - 80.0) | 0 - 100 | 0.92 | 0.95 |
| **Health: Role limitations due to physical health (RAND-36)** | 15.9 (32.2) | 0.0 (0.0 - 12.5) | 0 - 100 | 0.88 | 0.90 |
| **Health: Role limitations due to emotional problems (RAND-36)** | 45.5 (47.8) | 33.3 (0.0 - 100.0) | 0 - 100 | 0.93 | 0.90 |

Means (SD) and medians (Q1-Q3) are calculated for questionnaire sum scores, and the associated Cronbach's α across the corresponding individual questions. FSS, Fatigue Severity Scale; GAD-7, General Anxiety Disorder-7; ISI, Insomnia Severity Index; MFIS, Modified Fatigue Impact Scale; PHQ-9, Patient Health Questionnaire 9; WHODAS 2.0, WHO Disability Assessment Schedule 2.0; RAND-36, RAND 36-Item Short Form Health Survey.

**Table S3: Correlations between [^11^C]PK11195 DVRs and final and sub section questionnaire scores in long covid participants (whole brain, NAWM, amygdala, hippocampus and thalamus)**

| **Questionnaire** | **Whole brain DVR** | **NAWM DVR** | **Cortex gray matter DVR** | **Amygdala DVR** | **Hippocampus DVR** | **Thalamus DVR** |
| --- | --- | --- | --- | --- | --- | --- |
| **Quality of life (EuroHIS-8)** | 0.17 [-0.51 - 0.72] (0.64) | -0.09 [-0.68 - 0.57] (0.81) | 0.43 [-0.27 - 0.84] (0.21) | -0.76 [-0.94 - -0.25] **(0.01)** | -0.83 [-0.96 - -0.42] **(<0.01)** | -0.70 [-0.92 - -0.12] **(0.03)** |
| **Disability (WHODAS 2.0)** | 0.33 [-0.37 - 0.80] (0.35) | 0.41 [-0.30 - 0.82] (0.24) | 0.05 [-0.60 - 0.66] (0.88) | 0.54 [-0.14 - 0.87] (0.11) | 0.49 [-0.20 - 0.86] (0.15) | 0.31 [-0.40 - 0.79] (0.38) |
| **Disability: cognition (WHODAS 2.0)** | -0.14 [-0.71 - 0.54] (0.70) | -0.13 [-0.70 - 0.54] (0.72) | -0.26 [-0.77 - 0.44] (0.46) | 0.64 [0.02 - 0.91] **(0.04)** | 0.71 [0.14 - 0.92] **(0.02)** | 0.44 [-0.27 - 0.84] (0.21) |
| **Disability: social (WHODAS 2.0)** | 0.02 [-0.61 - 0.64] (0.95) | 0.21 [-0.48 - 0.74] (0.55) | -0.19 [-0.73 - 0.50] (0.60) | 0.63 [0.00 - 0.90] (0.05) | 0.57 [-0.10 - 0.88] (0.09) | 0.25 [-0.45 - 0.76] (0.49) |
| **Disability: getting around (WHODAS 2.0)** | -0.08 [-0.68 - 0.58] (0.83) | 0.08 [-0.58 - 0.68] (0.83) | -0.50 [-0.86 - 0.19] (0.14) | 0.53 [-0.15 - 0.87] (0.11) | 0.67 [0.07 - 0.91] **(0.03)** | 0.40 [-0.30 - 0.82] (0.25) |
| **Disability: self-care (WHODAS 2.0)** | 0.44 [-0.26 - 0.84] (0.20) | 0.44 [-0.26 - 0.84] (0.20) | -0.19 [-0.73 - 0.50] (0.61) | 0.50 [-0.19 - 0.86] (0.14) | 0.62 [-0.02 - 0.90] (0.06) | 0.44 [-0.26 - 0.84] (0.20) |
| **Disability: household activities (WHODAS 2.0)** | 0.19 [-0.50 - 0.73] (0.60) | 0.17 [-0.51 - 0.72] (0.64) | 0.12 [-0.55 - 0.70] (0.74) | 0.13 [-0.55 - 0.70] (0.72) | 0.00 [-0.63 - 0.63] (1.00) | -0.07 [-0.67 - 0.59] (0.85) |
| **Disability: work activities (WHODAS 2.0)** | 0.29 [-0.42 - 0.78] (0.42) | 0.42 [-0.28 - 0.83] (0.23) | 0.09 [-0.57 - 0.68] (0.81) | 0.53 [-0.15 - 0.87] (0.12) | 0.41 [-0.29 - 0.83] (0.23) | 0.32 [-0.39 - 0.79] (0.37) |
| **Disability: societal (WHODAS 2.0)** | 0.26 [-0.44 - 0.76] (0.47) | 0.43 [-0.27 - 0.83] (0.21) | -0.07 [-0.67 - 0.59] (0.85) | 0.61 [-0.03 - 0.90] (0.06) | 0.58 [-0.08 - 0.88] (0.08) | 0.42 [-0.29 - 0.83] (0.23) |
| **Fatigue (FSS)** | 0.21 [-0.44 - 0.72] (0.53) | 0.17 [-0.48 - 0.70] (0.61) | 0.13 [-0.51 - 0.68] (0.70) | 0.11 [-0.52 - 0.67] (0.74) | 0.35 [-0.31 - 0.79] (0.29) | 0.32 [-0.35 - 0.77] (0.35) |
| **Fatigue (MFIS)** | 0.30 [-0.37 - 0.76] (0.37) | 0.37 [-0.29 - 0.79] (0.26) | -0.03 [-0.62 - 0.58] (0.94) | 0.50 [-0.14 - 0.85] (0.12) | 0.75 [0.26 - 0.93] **(0.01)** | 0.68 [0.14 - 0.91] **(0.02)** |
| **Fatigue: cognitive (MFIS)** | 0.24 [-0.42 - 0.73] (0.48) | 0.23 [-0.43 - 0.73] (0.49) | 0.09 [-0.54 - 0.66] (0.79) | 0.50 [-0.14 - 0.85] (0.12) | 0.61 [0.02 - 0.89] (0.05) | 0.56 [-0.07 - 0.87] (0.08) |
| **Fatigue: physical (MFIS)** | 0.27 [-0.40 - 0.75] (0.43) | 0.32 [-0.35 - 0.77] (0.34) | 0.05 [-0.57 - 0.63] (0.89) | 0.22 [-0.44 - 0.72] (0.52) | 0.54 [-0.09 - 0.86] (0.09) | 0.48 [-0.17 - 0.84] (0.14) |
| **Fatigue: psychosocial (MFIS)** | 0.49 [-0.16 - 0.84] (0.13) | 0.49 [-0.15 - 0.84] (0.12) | 0.35 [-0.31 - 0.79] (0.28) | 0.06 [-0.56 - 0.64] (0.85) | 0.28 [-0.39 - 0.75] (0.41) | 0.33 [-0.33 - 0.78] (0.32) |
| **Anxiety (GAD-7)** | 0.05 [-0.60 - 0.66] (0.89) | 0.36 [-0.35 - 0.81] (0.31) | -0.26 [-0.76 - 0.45] (0.48) | 0.83 [0.41 - 0.96] **(<0.01)** | 0.88 [0.55 - 0.97] **(<0.01)** | 0.57 [-0.10 - 0.88] (0.09) |
| **Depression (PHQ-9)** | 0.07 [-0.59 - 0.67] (0.85) | 0.22 [-0.48 - 0.74] (0.55) | -0.29 [-0.78 - 0.42] (0.42) | 0.75 [0.22 - 0.94] **(0.01)** | 0.87 [0.53 - 0.97] **(<0.01)** | 0.59 [-0.06 - 0.89] (0.07) |
| **Sleep (ISI)** | 0.01 [-0.63 - 0.63] (0.99) | 0.23 [-0.47 - 0.75] (0.52) | -0.26 [-0.77 - 0.44] (0.46) | 0.49 [-0.20 - 0.85] (0.15) | 0.59 [-0.07 - 0.89] (0.08) | 0.27 [-0.43 - 0.77] (0.44) |
| **Health: General health (RAND-36)** | -0.26 [-0.74 - 0.40] (0.44) | -0.52 [-0.85 - 0.12] (0.10) | -0.04 [-0.63 - 0.57] (0.90) | -0.38 [-0.80 - 0.28] (0.24) | -0.57 [-0.87 - 0.04] (0.07) | -0.49 [-0.84 - 0.16] (0.13) |
| **Health: Energy/fatigue (RAND-36)** | -0.06 [-0.64 - 0.56] (0.85) | -0.24 [-0.73 - 0.42] (0.48) | 0.35 [-0.32 - 0.78] (0.29) | -0.37 [-0.79 - 0.30] (0.27) | -0.64 [-0.90 - -0.07] **(0.03)** | -0.50 [-0.85 - 0.14] (0.12) |
| **Health: Physical functioning (RAND-36)** | -0.05 [-0.63 - 0.57] (0.89) | -0.24 [-0.74 - 0.42] (0.47) | 0.33 [-0.34 - 0.78] (0.32) | -0.13 [-0.68 - 0.51] (0.71) | -0.50 [-0.85 - 0.14] (0.12) | -0.39 [-0.80 - 0.28] (0.24) |
| **Health: Pain (RAND-36)** | -0.16 [-0.69 - 0.49] (0.64) | -0.25 [-0.74 - 0.41] (0.46) | 0.11 [-0.52 - 0.67] (0.74) | -0.47 [-0.84 - 0.18] (0.14) | -0.68 [-0.91 - -0.13] **(0.02)** | -0.59 [-0.88 - 0.01] (0.05) |
| **Health: Emotional well-being (RAND-36)** | -0.16 [-0.69 - 0.49] (0.65) | -0.41 [-0.81 - 0.26] (0.21) | 0.18 [-0.47 - 0.70] (0.60) | -0.38 [-0.80 - 0.28] (0.24) | -0.65 [-0.90 - -0.08] **(0.03)** | -0.44 [-0.82 - 0.22] (0.18) |
| **Health: Role limitations due to physical health (RAND-36)** | -0.28 [-0.75 - 0.38] (0.40) | -0.39 [-0.80 - 0.27] (0.23) | -0.25 [-0.74 - 0.41] (0.46) | -0.30 [-0.76 - 0.37] (0.37) | -0.27 [-0.75 - 0.39] (0.42) | -0.40 [-0.81 - 0.26] (0.22) |
| **Health: Role limitations due to emotional problems (RAND-36)** | 0.00 [-0.60 - 0.60] (0.99) | -0.35 [-0.78 - 0.32] (0.30) | 0.20 [-0.46 - 0.71] (0.56) | -0.44 [-0.82 - 0.22] (0.18) | -0.55 [-0.87 - 0.07] (0.08) | -0.42 [-0.81 - 0.24] (0.20) |
| **Health: Social functioning (RAND-36)** | -0.40 [-0.81 - 0.26] (0.22) | -0.49 [-0.84 - 0.16] (0.13) | -0.17 [-0.70 - 0.48] (0.63) | -0.24 [-0.73 - 0.42] (0.49) | -0.41 [-0.81 - 0.25] (0.21) | -0.40 [-0.80 - 0.27] (0.23) |

Spearman correlations, 5% to 95% confidence intervals [0.95 - 0.05 CI] and naive two tailed t test p-values (p). FSS, Fatigue Severity Scale; GAD-7, General Anxiety Disorder-7; ISI, Insomnia Severity Index; MFIS, Modified Fatigue Impact Scale; PHQ-9, Patient Health Questionnaire 9; WHODAS 2.0, WHO Disability Assessment Schedule 2.0; RAND-36, RAND 36-Item Short Form Health Survey; NAWM, normal appearing white matter.

**Table S4: Correlations between [^11^C]PK11195 DVRs and final and sub section questionnaire scores in long covid participants (putamen, pallidum, cingulate cortex, corpus callosum and brain stem)**

| **Questionnaire** | **Putamen DVR** | **Pallidum DVR** | **Cingulate cortex DVR** | **Corpus callosum DVR** | **Brain stem DVR** |
| --- | --- | --- | --- | --- | --- |
| **Quality of life (EuroHIS-8)** | -0.51 [-0.86 - 0.17] (0.129) | -0.17 [-0.72 - 0.52] (0.648) | -0.38 [-0.81 - 0.33] (0.280) | 0.22 [-0.48 - 0.75] (0.541) | -0.13 [-0.70 - 0.54] (0.711) |
| **Disability (WHODAS 2.0)** | 0.53 [-0.14 - 0.87] (0.111) | 0.05 [-0.60 - 0.66] (0.881) | 0.27 [-0.44 - 0.77] (0.455) | 0.11 [-0.56 - 0.69] (0.763) | 0.13 [-0.55 - 0.70] (0.725) |
| **Disability: cognition (WHODAS 2.0)** | 0.33 [-0.38 - 0.79] (0.359) | 0.04 [-0.60 - 0.65] (0.906) | 0.44 [-0.27 - 0.84] (0.208) | -0.52 [-0.87 - 0.16] (0.122) | 0.09 [-0.57 - 0.68] (0.800) |
| **Disability: social (WHODAS 2.0)** | 0.34 [-0.37 - 0.80] (0.334) | 0.03 [-0.61 - 0.65] (0.933) | 0.21 [-0.49 - 0.74] (0.565) | -0.15 [-0.71 - 0.53] (0.674) | 0.53 [-0.15 - 0.87] (0.115) |
| **Disability: household activities (WHODAS 2.0)** | 0.07 [-0.59 - 0.67] (0.854) | -0.32 [-0.79 - 0.39] (0.372) | -0.16 [-0.72 - 0.52] (0.649) | 0.01 [-0.62 - 0.64] (0.973) | -0.09 [-0.68 - 0.57] (0.802) |
| **Disability: work activities (WHODAS 2.0)** | 0.50 [-0.19 - 0.86] (0.145) | 0.01 [-0.63 - 0.63] (0.986) | 0.16 [-0.52 - 0.72] (0.653) | 0.21 [-0.49 - 0.74] (0.566) | 0.35 [-0.36 - 0.80] (0.320) |
| **Disability: societal (WHODAS 2.0)** | 0.54 [-0.14 - 0.87] (0.108) | 0.10 [-0.56 - 0.69] (0.777) | 0.21 [-0.48 - 0.74] (0.556) | 0.18 [-0.51 - 0.73] (0.627) | 0.26 [-0.44 - 0.76] (0.467) |
| **Disability: self-care (WHODAS 2.0)** | 0.37 [-0.34 - 0.81] (0.299) | 0.20 [-0.49 - 0.74] (0.577) | 0.40 [-0.31 - 0.82] (0.258) | 0.05 [-0.60 - 0.66] (0.886) | 0.29 [-0.41 - 0.78] (0.415) |
| **Disability: getting around (WHODAS 2.0)** | 0.31 [-0.40 - 0.79] (0.382) | -0.11 [-0.69 - 0.56] (0.763) | 0.14 [-0.54 - 0.71] (0.699) | -0.12 [-0.70 - 0.55] (0.737) | -0.09 [-0.68 - 0.58] (0.815) |
| **Fatigue (FSS)** | 0.20 [-0.45 - 0.71] (0.554) | -0.05 [-0.63 - 0.56] (0.873) | -0.10 [-0.66 - 0.53] (0.769) | -0.38 [-0.80 - 0.28] (0.244) | -0.10 [-0.66 - 0.53] (0.779) |
| **Fatigue (MFIS)** | 0.53 [-0.11 - 0.86] (0.096) | 0.25 [-0.42 - 0.74] (0.467) | 0.26 [-0.40 - 0.75] (0.433) | -0.24 [-0.73 - 0.42] (0.484) | 0.16 [-0.48 - 0.70] (0.631) |
| **Fatigue: physical (MFIS)** | 0.38 [-0.29 - 0.80] (0.254) | 0.14 [-0.50 - 0.68] (0.687) | -0.01 [-0.61 - 0.59] (0.979) | -0.13 [-0.68 - 0.51] (0.707) | -0.06 [-0.63 - 0.56] (0.872) |
| **Fatigue: cognitive (MFIS)** | 0.54 [-0.09 - 0.86] (0.088) | 0.27 [-0.39 - 0.75] (0.416) | 0.39 [-0.27 - 0.80] (0.233) | -0.35 [-0.79 - 0.32] (0.290) | 0.25 [-0.42 - 0.74] (0.466) |
| **Fatigue: psychosocial (MFIS)** | 0.47 [-0.18 - 0.83] (0.145) | 0.12 [-0.52 - 0.67] (0.726) | -0.09 [-0.65 - 0.54] (0.798) | 0.24 [-0.42 - 0.74] (0.469) | 0.06 [-0.56 - 0.64] (0.861) |
| **Anxiety (GAD-7)** | 0.53 [-0.14 - 0.87] (0.111) | 0.22 [-0.47 - 0.75] (0.532) | 0.27 [-0.43 - 0.77] (0.444) | 0.03 [-0.61 - 0.65] (0.934) | 0.53 [-0.14 - 0.87] (0.111) |
| **Depression (PHQ-9)** | 0.39 [-0.31 - 0.82] (0.260) | 0.03 [-0.61 - 0.65] (0.933) | 0.27 [-0.43 - 0.77] (0.449) | -0.07 [-0.67 - 0.58] (0.839) | 0.23 [-0.47 - 0.75] (0.527) |
| **Sleep (ISI)** | 0.17 [-0.51 - 0.72] (0.637) | 0.27 [-0.43 - 0.77] (0.454) | 0.07 [-0.59 - 0.67] (0.854) | -0.09 [-0.68 - 0.58] (0.815) | 0.23 [-0.47 - 0.75] (0.531) |
| **Health: General health (RAND-36)** | -0.50 [-0.85 - 0.14] (0.117) | -0.16 [-0.70 - 0.48] (0.631) | 0.00 [-0.60 - 0.60] (1.000) | -0.09 [-0.66 - 0.54] (0.790) | -0.06 [-0.64 - 0.56] (0.853) |
| **Health: Energy/fatigue (RAND-36)** | -0.38 [-0.80 - 0.29] (0.255) | -0.01 [-0.61 - 0.59] (0.979) | -0.05 [-0.63 - 0.57] (0.894) | 0.22 [-0.43 - 0.73] (0.507) | -0.01 [-0.61 - 0.59] (0.968) |
| **Health: Emotional well-being (RAND-36)** | -0.44 [-0.82 - 0.22] (0.177) | -0.13 [-0.68 - 0.51] (0.708) | 0.06 [-0.56 - 0.64] (0.862) | 0.00 [-0.60 - 0.60] (0.989) | -0.28 [-0.75 - 0.38] (0.399) |
| **Health: Physical functioning (RAND-36)** | -0.32 [-0.77 - 0.35] (0.338) | 0.01 [-0.59 - 0.61] (0.979) | 0.15 [-0.50 - 0.69] (0.668) | -0.06 [-0.64 - 0.56] (0.852) | 0.21 [-0.45 - 0.72] (0.535) |
| **Health: Social functioning (RAND-36)** | -0.70 [-0.92 - -0.18] **(0.016)** | -0.30 [-0.76 - 0.37] (0.375) | -0.20 [-0.71 - 0.45] (0.554) | -0.19 [-0.71 - 0.46] (0.572) | -0.36 [-0.79 - 0.31] (0.282) |
| **Health: Pain (RAND-36)** | -0.55 [-0.86 - 0.08] (0.080) | -0.29 [-0.76 - 0.37] (0.382) | -0.34 [-0.78 - 0.33] (0.308) | 0.19 [-0.46 - 0.71] (0.581) | -0.07 [-0.64 - 0.55] (0.831) |
| **Health: Role limitations due to physical health (RAND-36)** | -0.55 [-0.86 - 0.08] (0.080) | -0.03 [-0.62 - 0.58] (0.933) | -0.02 [-0.61 - 0.59] (0.960) | -0.18 [-0.70 - 0.47] (0.598) | -0.05 [-0.63 - 0.57] (0.893) |
| **Health: Role limitations due to emotional problems (RAND-36)** | -0.57 [-0.87 - 0.05] (0.069) | -0.22 [-0.72 - 0.44] (0.525) | -0.01 [-0.61 - 0.59] (0.966) | -0.20 [-0.71 - 0.46] (0.564) | -0.19 [-0.71 - 0.47] (0.584) |

Spearman correlations, 5% to 95% confidence intervals [0.95 - 0.05 CI] and naive two tailed t test p-values (p). FSS, Fatigue Severity Scale; GAD-7, General Anxiety Disorder-7; ISI, Insomnia Severity Index; MFIS, Modified Fatigue Impact Scale; PHQ-9, Patient Health Questionnaire 9; WHODAS 2.0, WHO Disability Assessment Schedule 2.0; RAND-36, RAND 36-Item Short Form Health Survey; NAWM, normal appearing white matter.

**Table S5: Demographics of** **relapsing remitting MS and secondary progressive MS participants**

| **Parameter** | **HC** | **RRMS** | **SPMS** | **HC vs RRMS** | **HC vs SPMS** |
| --- | --- | --- | --- | --- | --- |
| **n** | 11 | 6 | 7 |  |  |
| **m/f** | 7/4 | 2/4 | 1/6 | 0.335 | 0.066 |
| **Age (years)** | 45.55 (12.16) | 43.58 (4.87) | 49.54 (6.97) | 0.645 (0.645) | 0.390 (0.645) |
| **Weight (kg)** | 78.90 (77.20 - 85.15) | 85.00 (84.25 - 112.97) | 78.00 (63.80 - 92.50) | 0.291 (0.582) | 0.717 (0.717) |
| **BMI (kg/m^2^)** | 26.91 (3.89) | 31.00 (10.81) | 29.61 (6.47) | 0.406 (0.406) | 0.345 (0.406) |
| **NfL (pg/ml)** | 8.14 (4.76) | 6.17 (2.23) | 13.08 (6.72) | 0.296 (0.296) | 0.125 (0.249) |
| **GFAP (pg/ml)** | 53.71 (44.41 - 63.17) | 65.62 (56.51 - 67.17) | 117.01 (80.95 - 147.91) | 0.310 (0.310) | 0.033 (0.066) |

For normally distributed parameters, mean (SD) and t-test p-values are reported; for non-normally distributed parameters, median (Q1-Q3) and Wilcoxon test p-values are reported. P-values in brackets are corrected for within parameter multiple comparisons using Benjamini-Hochberg method. MS, multiple sclerosis; HC, healthy control; RRMS, relapsing remitting MS; SPMS, secondary progressive; BMI, body weight index; NfL, neurofilament light chain; GFAP, glial fibrillary acidic protein.


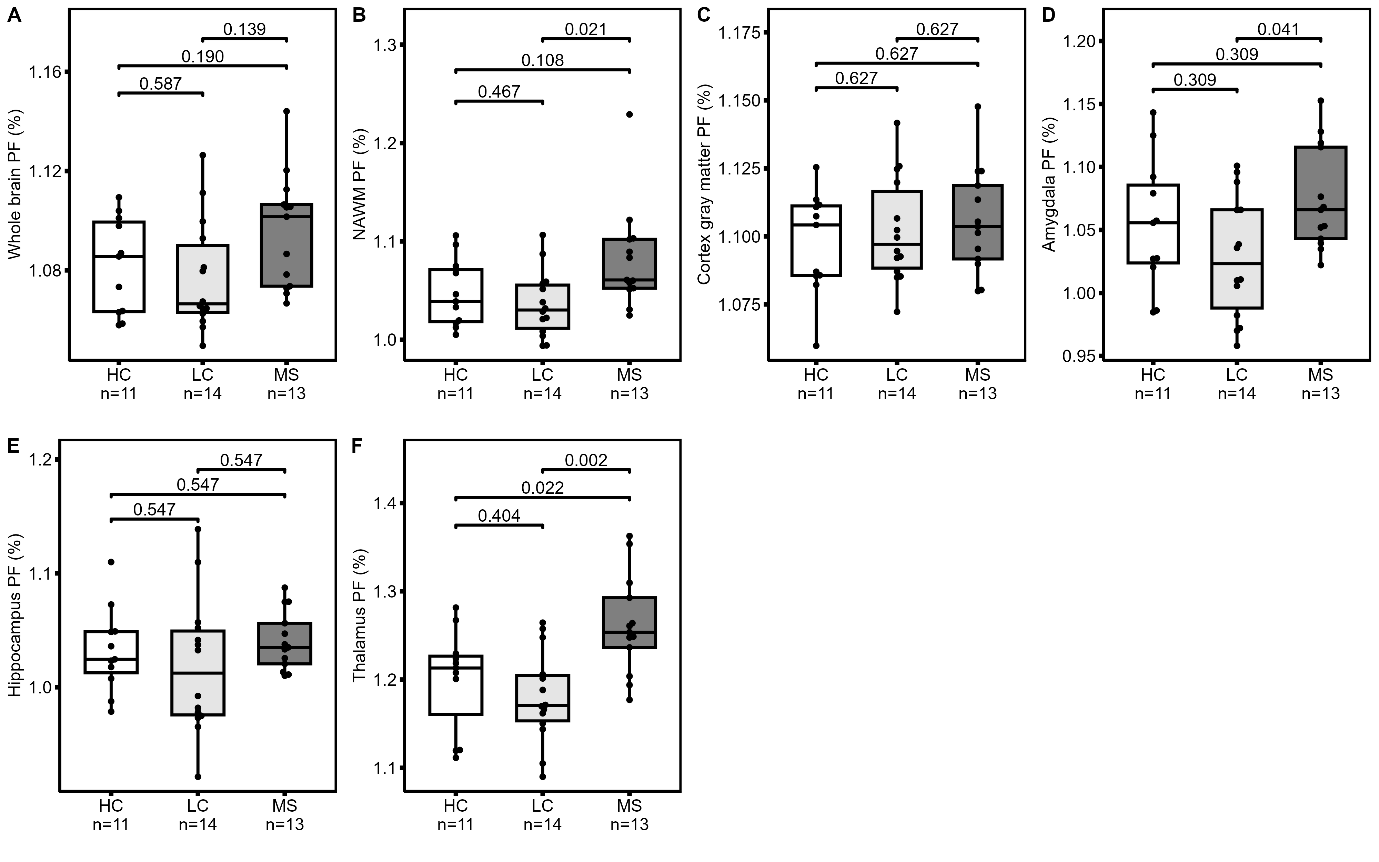


**Fig. S1** Regional brain volume parenchymal fractions in healthy control (HC), long covid (LC) and multiple sclerosis (MS) participants. Parenchymal fractions and within parameter multiple comparison corrected (Benjamini-Hochberg method) Welch’s t-test (**A,C-F**) or Wilcoxon test (**B**) p-values for group wise comparisons are reported. **A-F.** Whole brain, normal appearing white matter (NAWM), cortex gray matter, amygdala, hippocampus and thalamus parenchymal fractions

**Table S6: [^11^C]PK11195 DVRs and brain PFs in relapsing remitting MS and secondary progressive MS participants**

| **Parameter** | **RRMS** | **HC** | **SPMS** | **HC vs RRMS** | **HC vs SPMS** |
| --- | --- | --- | --- | --- | --- |
| **Whole brain DVR** | 1.10 (1.08 - 1.12) | 1.09 (1.06 - 1.10) | 1.10 (1.07 - 1.11) | 0.180 (0.211) | 0.211 (0.211) |
| **NAWM DVR** | 1.09 (0.08) | 1.05 (0.03) | 1.08 (0.02) | 0.303 (0.303) | **0.023 (0.045)** |
| **Cortex gray matter DVR** | 1.11 (0.02) | 1.10 (0.02) | 1.10 (0.02) | 0.153 (0.306) | 0.806 (0.806) |
| **Amygdala DVR** | 1.06 (0.03) | 1.05 (0.05) | 1.09 (0.04) | 0.835 (0.835) | 0.160 (0.319) |
| **Hippocampus DVR** | 1.05 (0.03) | 1.03 (0.04) | 1.04 (0.02) | 0.428 (0.808) | 0.808 (0.808) |
| **Thalamus DVR** | 1.26 (0.07) | 1.20 (0.06) | 1.27 (0.05) | 0.109 (0.109) | **0.022 (0.044)** |
| **Putamen DVR** | 1.15 (1.14 - 1.20) | 1.13 (1.11 - 1.17) | 1.19 (1.15 - 1.21) | 0.216 (0.216) | 0.126 (0.216) |
| **Pallidum DVR** | 1.15 (1.14 - 1.21) | 1.16 (1.11 - 1.19) | 1.19 (1.17 - 1.20) | 0.660 (0.660) | 0.189 (0.378) |
| **Cingulate cortex DVR** | 1.10 (0.04) | 1.09 (0.03) | 1.09 (0.04) | 0.612 (0.664) | 0.664 (0.664) |
| **Corpus callosum DVR** | 0.86 (0.84 - 0.91) | 0.91 (0.88 - 0.91) | 0.86 (0.85 - 0.90) | 0.660 (0.660) | 0.596 (0.660) |
| **Brain stem DVR** | 1.23 (0.05) | 1.20 (0.05) | 1.24 (0.05) | 0.177 (0.177) | 0.143 (0.177) |
| **Whole brain volume PF** | 81.69 (4.97) | 84.68 (3.11) | 82.75 (2.90) | 0.221 (0.221) | 0.201 (0.221) |
| **NAWM volume PF** | 31.93 (3.37) | 34.50 (2.12) | 32.14 (1.82) | 0.132 (0.132) | **0.024 (0.048)** |
| **Cortex gray matter volume PF** | 31.05 (1.94) | 32.35 (2.23) | 31.08 (2.32) | 0.233 (0.271) | 0.271 (0.271) |
| **Amygdala volume PF** | 0.23 (0.04) | 0.24 (0.02) | 0.24 (0.02) | 0.543 (0.802) | 0.802 (0.802) |
| **Hippocampus volume PF** | 0.59 (0.54 - 0.66) | 0.65 (0.63 - 0.66) | 0.58 (0.57 - 0.60) | 0.216 (0.216) | **0.020 (0.041)** |
| **Thalamus volume PF** | 0.93 (0.14) | 1.09 (0.07) | 0.93 (0.08) | **0.038 (0.038)** | **0.001 (0.002)** |
| **Putamen volume PF** | 0.61 (0.08) | 0.67 (0.07) | 0.57 (0.06) | 0.129 (0.129) | **0.007 (0.013)** |
| **Pallidum volume PF** | 0.26 (0.25 - 0.26) | 0.30 (0.27 - 0.30) | 0.26 (0.24 - 0.28) | **0.010 (0.021)** | **0.044 (0.044)** |
| **Cingulate cortex volume PF** | 1.31 (0.15) | 1.32 (0.19) | 1.29 (0.10) | 0.943 (0.943) | 0.651 (0.943) |
| **Corpus callosum volume PF** | 0.27 (0.05) | 0.27 (0.03) | 0.28 (0.04) | 0.944 (0.944) | 0.650 (0.944) |
| **Brain stem volume PF** | 1.38 (0.20) | 1.57 (0.06) | 1.47 (0.17) | 0.065 (0.130) | 0.193 (0.193) |

For normally distributed parameters mean (SD) and t-test p-values are reported; for non-normally distributed parameters, median (Q1-Q3) and Wilcoxon test p-values are reported. P-values in brackets are corrected for within parameter multiple comparisons using Benjamini-Hochberg method. MS, multiple sclerosis; HC, healthy control; RRMS, relapsing remitting MS; SPMS, secondary progressive MS; NAWM, normal appearing white matter; DVR, distribution volume ratio; PF, parenchymal fraction.

**Table S7. Demographic and soluble biomarker data of long COVID participants with SARS-CoV-2 diagnosis within 16 months of imaging versus over 16 months from imaging**

|  | **LC duration <= 16 months** | **LC duration > 16 months** | **LC <= 16 months vs**  **LC > 16 months** |
| --- | --- | --- | --- |
| **n** | 8 | 6 |  |
| **m/f** | 5/3 | 3/3 | 1.000 |
| **Age (years)** | 45.00 (9.68) | 45.00 (4.98) | 1.000 |
| **Weight (kg)** | 100.53 (27.74) | 103.12 (27.84) | 0.866 |
| **BMI (kg/m^2^)** | 31.05 (5.15) | 34.85 (9.43) | 0.401 |
| **NfL (pg/ml)** | 8.56 (7.85) | 7.82 (3.25) | 0.824 |
| **GFAP (pg/ml)** | 67.67 (46.48 - 87.52) | 72.39 (55.28 - 92.27) | 0.731 |
| **Long COVID duration (months)** | 10.62 (3.16) | 26.17 (5.00) | **<0.001** |

For normally distributed parameters, mean (SD) and t-test p-values are reported; for non-normally distributed parameters median (Q1-Q3) and Wilcoxon test p-values are reported. LC, long covid; BMI, body weight index; NfL, neurofilament light chain; GFAP, glial fibrillary acidic protein.

**Table S8. [^11^C]PK11195 DVRs and brain PFs of long COVID participants with SARS-CoV-2 diagnosis within 16 months of imaging versus over 16 months from imaging**

|  | **LC duration ≤ 16 months** | **LC duration > 16 months** | **LC ≤ 16 months vs**  **LC > 16 months** |
| --- | --- | --- | --- |
| **n** | 8 | 6 |  |
| **m/f** | 5/3 | 3/3 | 1.000 |
| **Whole brain DVR** | 1.08 (1.07 - 1.09) | 1.06 (1.06 - 1.06) | 0.059 |
| **NAWM DVR** | 1.05 (0.03) | 1.02 (0.02) | **0.042** |
| **Cortex gray matter DVR** | 1.10 (0.02) | 1.10 (0.02) | 0.761 |
| **Amygdala DVR** | 1.03 (0.05) | 1.03 (0.05) | 0.921 |
| **Hippocampus DVR** | 1.03 (0.07) | 1.01 (0.04) | 0.514 |
| **Thalamus DVR** | 1.19 (0.06) | 1.17 (0.05) | 0.534 |
| **Putamen DVR** | 1.13 (0.05) | 1.11 (0.03) | 0.554 |
| **Pallidum DVR** | 1.14 (0.05) | 1.10 (0.04) | 0.101 |
| **Cingulate cortex DVR** | 1.09 (0.03) | 1.09 (0.03) | 0.814 |
| **Corpus callosum DVR** | 0.88 (0.04) | 0.85 (0.03) | 0.195 |
| **Brain stem DVR** | 1.18 (0.06) | 1.17 (0.04) | 0.727 |
| **Whole brain volume PF** | 85.02 (2.25) | 86.94 (1.88) | 0.108 |
| **NAWM volume PF** | 33.95 (33.75 - 35.34) | 35.45 (35.09 - 37.15) | 0.108 |
| **Cortex gray matter volume PF** | 32.68 (1.94) | 32.86 (0.93) | 0.821 |
| **Amygdala volume PF** | 0.24 (0.03) | 0.24 (0.04) | 0.868 |
| **Hippocampus volume PF** | 0.60 (0.59 - 0.64) | 0.69 (0.62 - 0.71) | 0.345 |
| **Thalamus volume PF** | 1.06 (0.08) | 1.14 (0.09) | 0.136 |
| **Putamen volume PF** | 0.66 (0.07) | 0.67 (0.06) | 0.922 |
| **Pallidum volume PF** | 0.27 (0.02) | 0.29 (0.02) | 0.077 |
| **Cingulate cortex volume PF** | 1.32 (0.15) | 1.36 (0.11) | 0.584 |
| **Corpus callosum volume PF** | 0.28 (0.02) | 0.28 (0.03) | 0.784 |
| **Brain stem volume PF** | 1.54 (0.14) | 1.61 (0.10) | 0.263 |

For normally distributed parameters mean (SD) and t-test p-values are reported; for non-normally distributed parameters, median (Q1-Q3) and Wilcoxon test p-values are reported. LC, long covid; NAWM, normal appearing white matter; DVR, distribution volume ratio; PF, parenchymal fraction.
